# Analysis of Susceptibility and Drug Resistance of Antifungal Agents in Aspergillosis and Mucormycosis Patients: A Systematic Review

**DOI:** 10.1111/myc.70118

**Published:** 2025-10-18

**Authors:** Yinggai Song, Paul E. Verweij, Jochem B. Buil, Sybren de Hoog, Jie Liu, Jiaxian Guo, Wei Liu, Ruoyu Li

**Affiliations:** ^1^ Department of Dermatology Peking University First Hospital；National Clinical Research Center for Skin and Immune Diseases; Research Center for Medical Mycology, Peking University Beijing China; ^2^ Department of Medical Microbiology Radboudumc‐CWZ Expertise Center for Mycology Radboudumc Community for Infectious Diseases (RCI), Radboud University Medical Center Nijmegen the Netherlands; ^3^ Value & Implementation, Global Medical & Scientific Affairs, MSD China Shanghai China

**Keywords:** antifungal agents, *Aspergillus*, Mucorales, resistance, susceptibility

## Abstract

**Objectives:**

To evaluate the susceptibility and resistance of *Aspergillus* and Mucorales isolates to antifungal agents.

**Methods:**

Studies in susceptibility or resistance of *Aspergillus* and Mucorales isolates to antifungal agents published between January 2010 and June 2023 were systematically searched in PubMed, EMBASE and the Cochrane Library. The minimum inhibitory concentration (MIC), susceptibility and resistance data were analysed using CLSI or EUCAST methods.

**Results:**

After following the systematic review processes, 96 studies were included. The total number of isolates was 16,258. Compared with existing MIC distributions and breakpoints or epidemiological cutoff values (ECVs) established by CLSI or EUCAST, for *A. flavus* , the posaconazole and voriconazole MIC values were at or below the ECV, indicating that the isolates were wild‐type (WT) strains; however, the amphotericin B, isavuconazole and itraconazole MIC values were elevated. For *A. fumigatus* , the isavuconazole MIC values were within ECV limits, indicating that the isolates were WT strains; however, the amphotericin B, posaconazole and voriconazole MIC values were elevated. For *A. niger* , the isavuconazole and voriconazole MIC values were within ECV limits, indicating that the isolates were WT strains; however, the amphotericin B and posaconazole MIC values were elevated. *A. flavus* had consistently high in vitro susceptibility to voriconazole, and *A. fumigatus* and *A. niger* had consistently high in vitro susceptibility to amphotericin B. For Mucorales, the resistance to amphotericin B was consistently at the lowest level. The subgroup analysis indicated that the resistance among the strains in the environment was higher than that of the clinical isolates.

**Conclusion:**

Trends in susceptibility and resistance of *Aspergillus* and Mucorales isolates should be adequately considered in antifungal therapy. The evaluation of drug resistance is beneficial in that it enables clinicians to choose suitable drugs and appropriate doses.

## 1 Introduction

Invasive fungal diseases, especially aspergillosis and mucormycosis, are associated with high morbidity and mortality among a wide variety of immunocompromised patients, such as those receiving solid organ or stem cell transplants, those with haematological malignancies and those taking immunosuppressive agents [1]. It is estimated that over 2,113,000 people develop invasive aspergillosis each year globally [2]. The mortality rate for aspergillosis ranges from 30% to 90%, with the rate depending on the patient population and the severity and duration of immunosuppression [3]. The incidence of mucormycosis is rising globally. The Leading International Fungal Education (LIFE) portal has estimated that the annual prevalence of mucormycosis has increased to 910,000 cases globally [4]. All‐cause mortality rates for mucormycosis range considerably from 40% to 80% and vary with the underlying condition and site of infection [5]. The landscape of invasive fungal diseases is progressively changing. A successful clinical outcome generally requires early diagnosis and effective antifungal therapy. However, antifungal options for aspergillosis and mucormycosis are few, with the chemical classes available for treating invasive diseases limited to azoles (isavuconazole, itraconazole, posaconazole, voriconazole) and polyenes (amphotericin B).

Over the past decade, due to the expansion of antifungal use for prophylaxis and empirical and directed therapy in patients with various conditions, the epidemiology of invasive fungal infections has changed, which has led to increased drug resistance [6]. Additionally, the use of medically related antifungal drugs in agriculture has contributed to environmental reservoirs for some drug‐resistant pathogens [7, 8]. There is increasing concern about the global emergence of antifungal resistance among both clinical and environmental isolates, for which optimal therapies are not well defined. Factors related to antifungal agents, such as in vitro susceptibility and drug resistance, have an important bearing on the ultimate outcome of treatment and can help to predict the clinical response to therapy. In addition, the susceptibility of *Aspergillus* and Mucorales isolates to antifungal agents varies geographically.

Currently, there is no systematic review or meta‐analysis on the susceptibility and resistance of *Aspergillus* and Mucorales isolates to antifungal agents in aspergillosis and mucormycosis patients, despite the large amount of data in the literature. A better understanding of the clinical impact of antifungal resistance is essential for the prompt and efficient treatment of patients with aspergillosis and mucormycosis and for improving the outcome of such infections. As reported in this article, we systematically collected susceptibility and drug resistance data of *Aspergillus* isolates (specifically *Aspergillus fumigatus* , *Aspergillus flavus* and *Aspergillus niger*) and Mucorales isolates (specifically *Rhizopus* spp., *Mucor* spp. and *Rhizomucor* spp.) for different types of antifungal agents (specifically amphotericin B, isavuconazole, itraconazole, posaconazole and voriconazole).

## 2 Methods

This study followed the Preferred Reporting Items for Systematic Reviews and Meta‐Analyses (PRISMA 2020) extension for scoping reviews [9]. The protocol was registered on Inplasy (registration number: INPLASY202470069).

### 2.1 Data Sources and Literature Search

An electronic database search for PubMed, EMBASE and the Cochrane Library was conducted on June 13, 2023. The included studies focused on antifungal susceptibility and were published between January 2010 and June 2023. The search strategy is provided in S1.

### 2.2 Study Selection

The studies comprised in vitro studies that reported the susceptibility data of *Aspergillus* and Mucorales isolates to antifungal agents used to treat aspergillosis and mucormycosis. Moreover, molecular tests must be performed before the analysis according to standardised classification for fungus. Articles that did not use molecular tests will be excluded. Detailed inclusion criteria were as follows:

1. Population: Studies on *A. fumigatus* , *A. flavus* or *A. niger* and studies on *Rhizopus* spp., *Mucor* spp. or *Rhizomucor* spp.
2. Intervention: Studies on antifungal agents including amphotericin B, isavuconazole, itraconazole, posaconazole and voriconazole.
3. Comparator: no limitation.
4. Outcome: susceptibility, drug resistance and minimum inhibitory concentration (MIC) determined by the method of broth dilution.
5. Study design: In vitro studies.

### 2.3 Data Extraction

Data from each study was extracted independently by two reviewers using a standardised data extraction form. Any disagreements were resolved by discussion, with assistance from a third party if necessary. Where information relating to a potentially included study was lacking, we contacted the authors and requested further information. We extracted all relevant characteristics of the included studies, including:

(1) General study characteristics: First author's name, publication year, country, study design, funding sources.

(2) Pathogens: Type, sources (environmental or clinical).

(3) Interventions: Types of antifungal agents (amphotericin B, isavuconazole, itraconazole, posaconazole and voriconazole).

(4) Outcomes: Susceptibility, drug resistance and MIC value (MIC range and MIC50).

### 2.4 Data Synthesis and Analysis

We summarised the susceptibility and resistance data of *Aspergillus* and Mucorales isolates to antifungal agents. The MIC, susceptibility and resistance data were presented with the median and range. When clinical breakpoint values were proposed based on the Clinical & Laboratory Standards Institute (CLSI) or the European Committee on Antimicrobial Susceptibility Testing (EUCAST) guidelines, MIC values were interpreted in accordance with them. The MIC data in our study were compared with the cutoff values proposed by CLSI or EUCAST guidelines, and the therapy outcome was predicted based on these results. In the absence of clinical breakpoints, in accordance with Astvad 2022 [10], after ascertaining that the species was correctly identified, we compared the MIC values with existing MIC distributions for that drug and species to determine whether the MIC was ‘normal’ or ‘elevated’. If the MIC values were at or below the epidemiological cutoff value, the isolates were defined as wild‐type (WT).

This is a narrative evidence synthesis, and we performed subgroup analyses in the following categories: (1) susceptibility and drug resistance data between environmental and clinical isolates; (2) susceptibility and drug resistance data in different patient populations.

## 3 Results

### 3.1 Results of Study Selection

**FIGURE 1:**
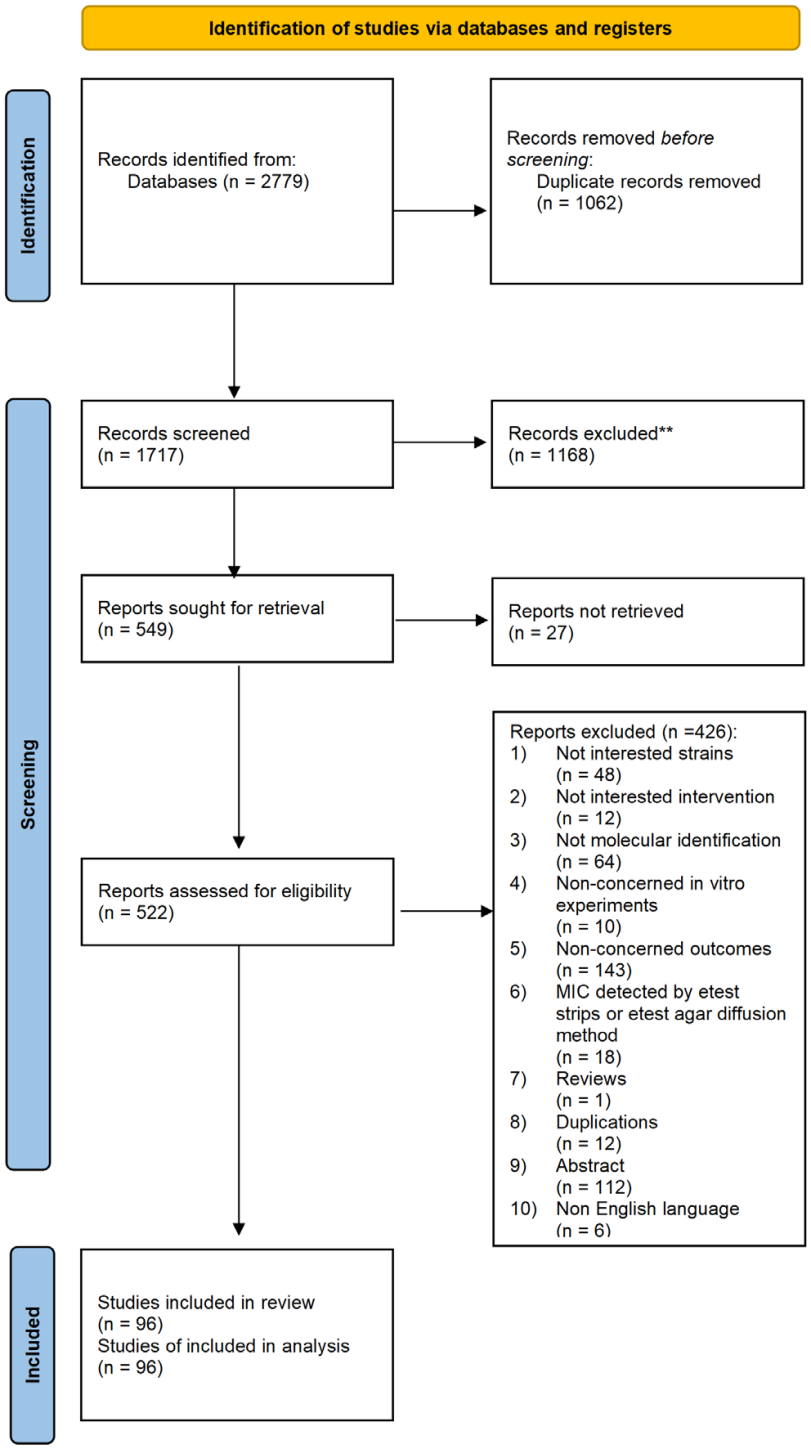
Flow of study review and selection process.

The trial search identified 2779 records, 1717 of which remained after removing duplicates. Next, 1168 records were excluded after screening the title and abstract. Subsequently, 27 reports were not retrieved, leaving 522 reports with the full text to be assessed for eligibility. Finally, 426 reports were excluded following screening of the full text, leaving the 96 studies [11, 12, 13, 14, 15, 16, 17, 18, 19, 20, 21, 22, 23, 24, 25, 26, 27, 28, 29, 30, 31, 32, 33, 34, 35, 36, 37, 38, 39, 40, 41, 42, 43, 44, 45, 46, 47, 48, 49, 50, 51, 52, 53, 54, 55, 56, 57, 58, 59, 60, 61, 62, 63, 64, 65, 66, 67, 68, 69, 70, 71, 72, 73, 74, 75, 76, 77, 78, 79, 80, 81, 82, 83, 84, 85, 86, 87, 88, 89, 90, 91, 92, 93, 94, 95, 96, 97, 98, 99, 100, 101, 102, 103, 104, 105, 106] included in this review. The study‐screening process and the reasons for exclusion at the full‐text screening stage are presented in Figure 1.

### 3.2 Summary of Characteristics of Included Studies

We included 96 studies from multiple countries or regions. The total number of isolates was 16,258. The strains isolated from patients, or the environment were *A. fumigatus* , *A. flavus* , *A. niger* , *Rhizopus* spp., *Mucor* spp. and *Rhizomucor* spp. Strains were isolated from patients in 79 studies (including 14,993 isolates), the environment in five studies (including 269 isolates), both patients and the environment in 8 studies (including 971 isolates), and the laboratory in two studies. One study did not report the strain source. To determine the MIC, 76 studies used the CLSI method, 18 used the EUCAST method, one used both methods and two did not report the method. The general characteristics of the included studies, including the MIC determination methods, are summarised in Table S1.

### 3.3 MIC50 of Antifungal Agents for *Aspergillus* Isolates

**FIGURE 2:**
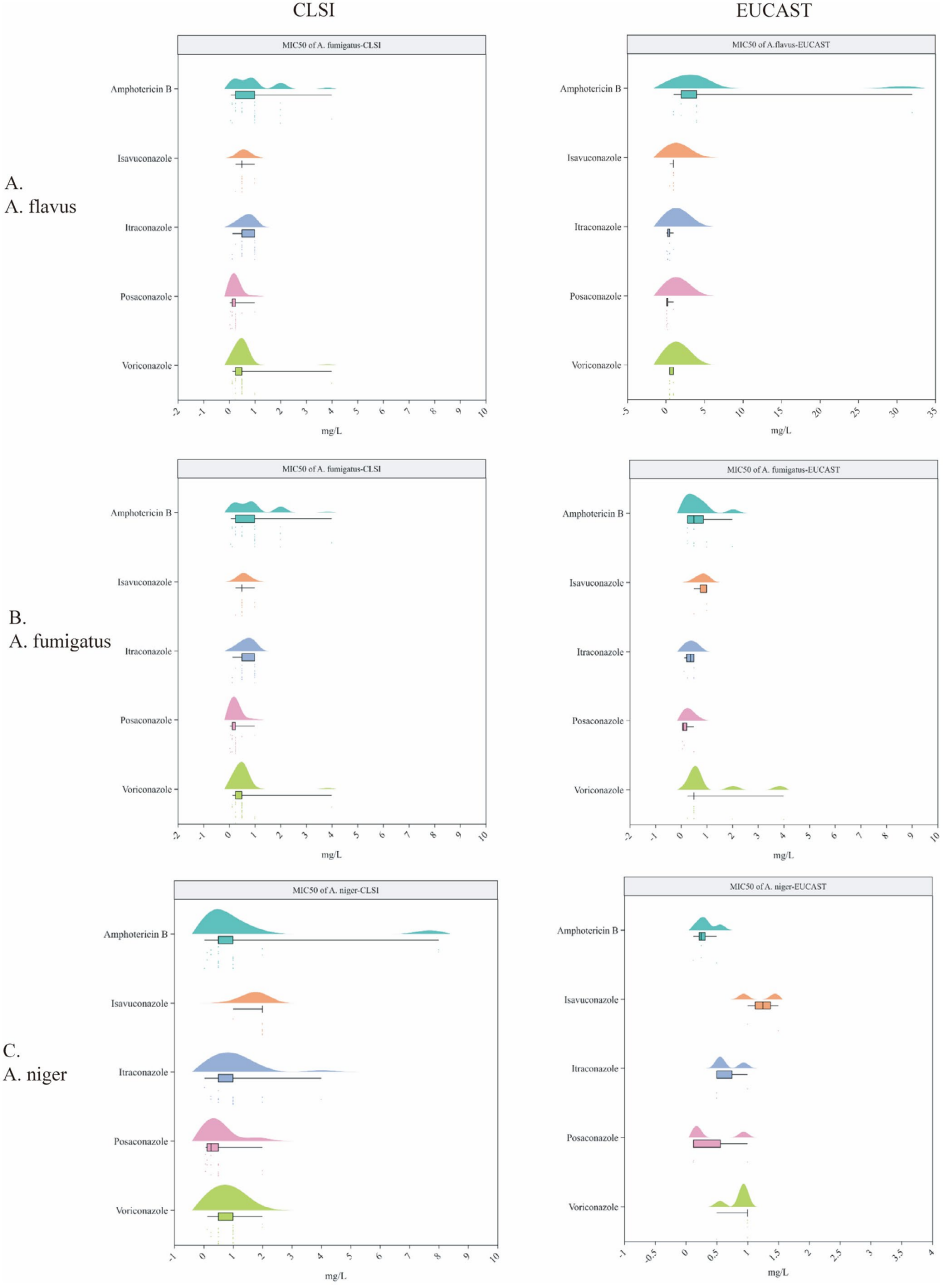
(A) MIC50 (median and range, mg/L) of antifungal agents for *Aspergillus* *flavus*; (B) MIC50 (median and range, mg/L) of antifungal agents for *Aspergillus fumigatus*; (C) MIC50 (median and range, mg/L) of antifungal agents for *Aspergillus niger*. *A. flavus* , *Aspergillus flavus*; *A. fumigatus* , *Aspergillus fumigatus* ; *A. niger* , *Aspergillus niger*; CLSI, Clinical and Laboratory Standards Institute; EUCAST, European Committee on Antimicrobial Susceptibility Testing; MIC50, 50% minimun inhibitory concentration.

For *A. flavus* , the MIC50 values (CLSI method) were as follows: amphotericin B 1 (0.125–4) mg/L (21 studies), isavuconazole 1 (0.5–2) mg/L (nine studies), itraconazole 0.375 (0.12–16) mg/L (20 studies), posaconazole 0.25 (0.12–0.5) mg/L (15 studies) and voriconazole 0.5 (0.125–1) mg/L (21 studies). The MIC50 values using the EUCAST method were higher for amphotericin B, ranging from 1 to 32 mg/L (seven studies); those of isavuconazole (five studies), itraconazole (seven studies), posaconazole (seven studies) and voriconazole (nine studies) obtained using the EUCAST method were 1 (0.5–1), 0.25 (0.12–1), 0.125 (0.12–1) and 0.5 (0.5–1) mg/L, respectively (Figure 2A and Table S2). Based on epidemiological cutoff values (ECVs) established by CLSI for *A. flavus* , the ECVs for WT strains were as follows: amphotericin B (ECVs WT ≤ 4), isavuconazole (ECVs WT ≤ 1), itraconazole (ECVs WT ≤ 1), posaconazole (ECVs WT ≤ 0.5), voriconazole (ECVs WT ≤ 2) [107]. Based on antifungal epidemiological cutoff values (ECOFFs) and clinical breakpoints (mg/L) established by EUCAST for *A. flavus* , the ECOFFs and breakpoints were amphotericin B (ECOFFs WT ≤ 4), isavuconazole (ECOFFs WT ≤ 2, *S* [susceptible] ≤ 1, *R* [resistant] > 2, ATU [area of technical uncertainty] = 2), itraconazole (ECOFFs WT ≤ 1, *S* ≤ 1, *R* > 1, ATU = 2), posaconazole (ECOFFs WT ≤ 0.5) and voriconazole (ECOFFs WT ≤ 2) [108]. Overall, for *A. flavus* , the MIC values of posaconazole and voriconazole were at or below the ECV (≤ 0.5 mg/L for posaconazole, ≤ 2 mg/L for voriconazole); thus, it can be assumed that the isolates were WT strains. However, the MIC values of amphotericin B, isavuconazole and itraconazole exceeded the ECV (≤ 4 mg/L for amphotericin B, ≤ 1 or 2 mg/L for isavuconazole, ≤ 1 mg/L for itraconazole) and were elevated compared with existing MIC distributions.

For *A. fumigatus* , the CLSI method yielded MIC50 values for amphotericin B (26 studies), isavuconazole (10 studies), itraconazole (29 studies), posaconazole (25 studies) and voriconazole (32 studies) of 1 (0.0625–4) mg/L, 0.5 (0.25–1) mg/L, 0.5 (0.125–1) mg/L, 0.25 (0.03–1) mg/L and 0.5 (0.125–4) mg/L, respectively. Using the EUCAST method, the MIC50 values were 0.5 (0.25–2) mg/L, 1 (0.5–1) mg/L, 0.375 (0.125–0.5) mg/L, 0.0925 (0.06–0.5) mg/L and 0.5 (0.25–4) mg/L, respectively (Figure 2B and Table S2). CLSI ECVs for *A. fumigatus* indicated WT cutoffs for amphotericin B, isavuconazole and itraconazole of ≤ 2, ≤ 1 and ≤ 1 mg/L, respectively. EUCAST ECOFFs and breakpoints showed slight variations, with WT cutoffs of ≤ 1 mg/L for amphotericin B, ≤ 2 mg/L for isavuconazole, ≤ 1 for itraconazole, ≤ 0.25 for posaconazole and ≤ 1 for voriconazole. MICs for isavuconazole and itraconazole were within ECV limits, so that the isolates were WT strains. However, the MICs for amphotericin B, posaconazole and voriconazole exceeded the established ECVs.

For *A. niger* , the MIC50 values (CLSI method) were as follows: amphotericin B 0.5 (0.0312–8) mg/L (19 studies), isavuconazole 2 (1–2) mg/L (six studies), itraconazole 1 (0.032–4) mg/L (17 studies), posaconazole 0.25 (0.063–2) mg/L (12 studies) and voriconazole 1 (0.125–2) mg/L (19 studies); the corresponding values obtained with the EUCAST method were 0.25 (0.12–0.5), 1.25 (1–1.5), 0.5 (0.5–1), 0.125 (0.12–1) and 1 (0.5–1) mg/L, respectively (Figure 2C and Table S2). CLSI ECVs for *A. niger* showed WT cutoffs for amphotericin B, isavuconazole, itraconazole, posaconazole and voriconazole of ≤ 2, ≤ 4, ≤ 4, ≤ 2 and ≤ 2 mg/L, respectively [107]. EUCAST ECOFFs and clinical breakpoints indicated some variations, with WT cutoffs of ≤ 0.5 mg/L for amphotericin B, ≤ 4 mg/L for isavuconazole, ≤ 2 for itraconazole, ≤ 0.5 for posaconazole and ≤ 2 for voriconazole [108]. The MIC values for isavuconazole, itraconazole and voriconazole were at or below the ECV; thus, it can be assumed that the isolates were WT strains. The MIC values for amphotericin B and posaconazole exceeded the ECV and were elevated compared with existing MIC distributions.

### 3.4 MIC50 of Antifungal Agents for Mucorales Isolates

**FIGURE 3:**
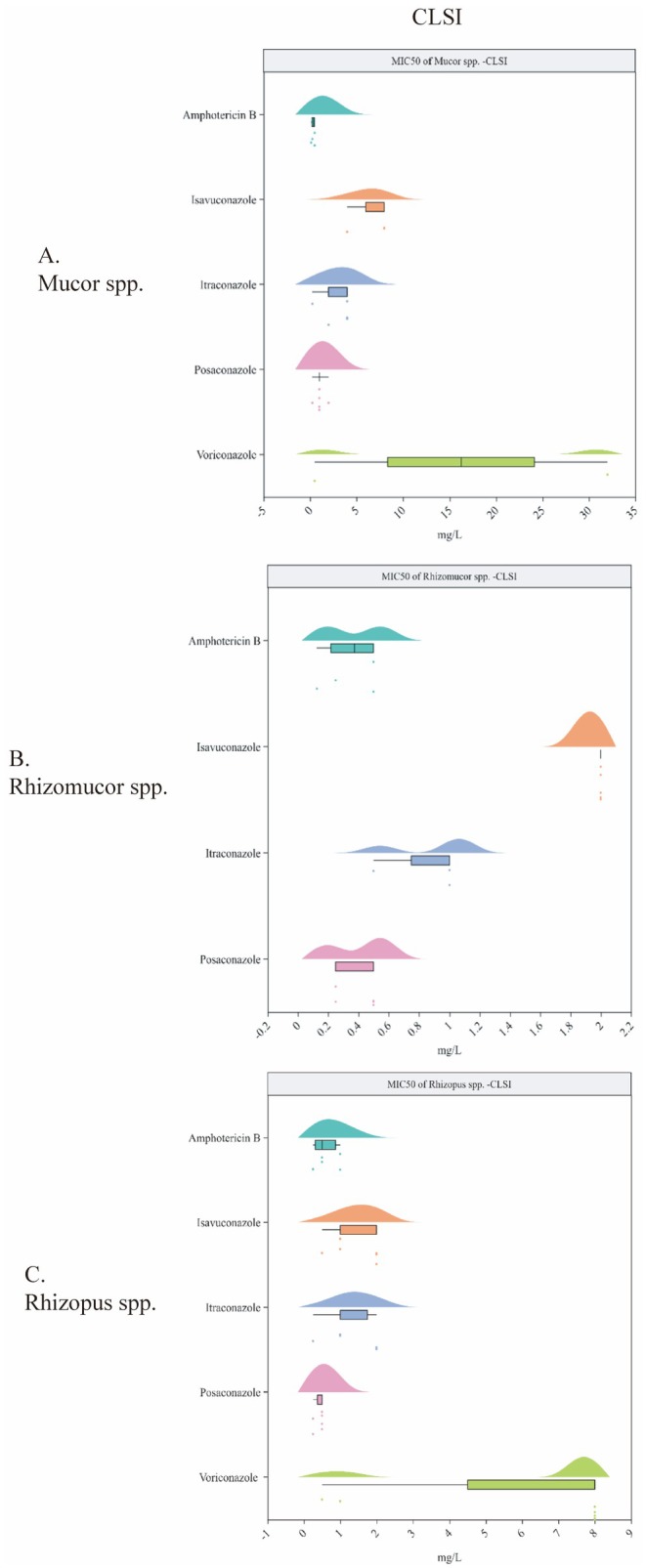
(A) MIC50 (median and range, mg/L) of antifungal agents for *Mucor*species; (B) MIC50 (median and range, mg/L) of antifungal agents for *Rhizomoucor* species; (C) MIC50 (median and range, mg/L) of antifungal agents for *Rhizopus* species. *Mucor spp*., *Mucor* species; *Rhizomucor spp*., *Rhizomucor* species; *Rhizopus spp., Rhizopus species.* CLSI, Clinical and Laboratory Standards Institute; MIC50, 50% minimun inhibitory concentration.

For *Mucor* spp., the MIC50 values of amphotericin B (4 studies), isavuconazole (five studies), itraconazole (five studies), posaconazole (six studies) and voriconazole (five studies), based on the CLSI method, were 0.375 (0.125–0.5), 8 (4–8), 4 (0.25–4), 1 (0.25–2) and 16.25 (0.5–32), respectively (Figure 3A and Table S3). No breakpoints or ECVs have been established for Mucorales, making it challenging to assess WT and non‐WT classifications from this data.

For *Rhizomucor* spp., the MIC50 values (CLSI method) of amphotericin B (three studies), isavuconazole (four studies), itraconazole (two studies) and posaconazole (four studies), voriconazole (2 studies) were 0.375 (0.125–0.5), 2 (2), 0.5 (0.5–1), 0.5 (0.25–0.5) and 2 mg/L, respectively (Figure 3B and Table S3).

For *Rhizopus* spp., the MIC50 values (CLSI method) of amphotericin B (five studies), isavuconazole (five studies), itraconazole (five studies), posaconazole (six studies) and voriconazole (six studies) were 0.5 (0.25–1), 1 (0.5–2), 1 (0.25–2), 0.5 (0.25–0.5) and 8 (0.5–8) mg/L, respectively (Figure 3C and Table S3).

### 3.5 Susceptibility to Antifungal Agents of *Aspergillus* Isolates

For *A. flavus* , the susceptibility to amphotericin B (12 studies), isavuconazole (four studies), itraconazole (10 studies), posaconazole (10 studies) and voriconazole (reported in 10 studies), based on the CLSI method, was 96.75% (6.5%–100%), 97.9% (83.2%–100%), 100% (7.1%–100%), 97.7% (59.7%–100%) and 100% (95.8%–100%); the corresponding values for amphotericin B (three studies), isavuconazole (two studies), itraconazole (four studies), posaconazole (four studies) and voriconazole (three studies), using the EUCAST method, were 96% (30%–100%), 98.45% (98%–98.9%), 98.55% (80%–100%), 99.75% (35%–100%) and 100% (60%–100%), respectively (Figure S1A and Table S4). The resistance is provided in Figure S2A and Table S5. The data indicate that *A. flavus* has been consistently high in vitro susceptible to voriconazole.

For *A. fumigatus* , the susceptibility (CLSI method) to amphotericin B (12 studies), isavuconazole (five studies), itraconazole (15 studies), posaconazole (10 studies) and voriconazole (16 studies) was 100% (95.7%–100%), 95.1% (88.7%–96.2%), 93.7% (83.3%–100%), 98% (79.4%–100%) and 96.8% (83.3%–100%); the corresponding values (EUCAST method) for amphotericin B (two studies), isavuconazole (one study), itraconazole (three studies), posaconazole (two studies) and voriconazole (reported in two studies) were 100% (100%), 91.7% (91.7%), 66.7% (52–100%), 33.3% (33.3%) and 100% (100%), respectively (Figure S1B and Table S4). The resistance is provided in Figure S2B and Table S5. The results indicate that *A. fumigatus* had consistently high in vitro susceptibility to amphotericin B.

For *A. niger* , the susceptibility (CLSI method) to amphotericin B (six studies), isavuconazole (three studies), itraconazole (seven studies), posaconazole (seven studies) and voriconazole (eight studies) was 100% (100%), 97.75% (95.7%–100%), 96.9% (86%–100%), 100% (11%–100%) and 98.05% (71%–100%); the corresponding values (EUCAST method) for amphotericin B (one study), itraconazole (one study), posaconazole (one study) and voriconazole (one study) were 80%, 67.5%, 50% and 87.5%, respectively (Figure S1C and Table S4). The resistance is provided in Figure S2C and Table S5. The data suggest that *A. niger* had consistently high in vitro susceptibility to amphotericin B, which had low resistance to the drug.

### 3.6 Susceptibility to Antifungal Agents Against Mucorales Isolates

There was no study reported drug sensitivity in *Mucor* spp., *Rhizopus* spp. or *Rhizomucor* spp. For *Mucor* spp., a single study reported resistance to amphotericin B, isavuconazole, itraconazole, posaconazole and voriconazole of 1.0%, 100.0%, 58.0%, 80.6% and 97.7%, respectively, based on the CLSI method (Table S6). For *Rhizomucor* spp., a single study reported resistance (CLSI method) to amphotericin B, isavuconazole, itraconazole, posaconazole and voriconazole of 0.0%, 100.0%, 18.8%, 51.4% and 87.5%, respectively (Table S6). For *Rhizopus* spp., a single study reported resistance (CLSI method) to amphotericin B, isavuconazole, itraconazole, posaconazole and voriconazole of 0.8%, 74.3%, 39.8%, 80.0% and 100.0%, respectively (Table S6). For *Rhizopus* spp., a single study reported resistance (EUCAST method) to amphotericin B and posaconazole of 29.4% and 17.6%, respectively (Table S6).

### 3.7 MIC Ranges of Antifungal Agents for *Aspergillus* Isolates

For *A. flavus* , the MIC ranges (CLSI method) were as follows: amphotericin B 0.0125–32 mg/L (32 studies), isavuconazole 0.06–16 mg/L (13 studies), itraconazole 0.03–64 mg/L (31 studies), posaconazole 0.03–4 mg/L (29 studies) and voriconazole 0.03–16 mg/L (33 studies); the corresponding ranges (EUCAST method) were as follows: amphotericin B 0.5–32 mg/L (10 studies), isavuconazole 0.125–16 mg/L (eight studies), itraconazole 0.031–16 mg/L (nine studies), posaconazole 0.03–4 mg/L (9 studies) and voriconazole 0.063–16 mg/L (11 studies) (Table S7).

For *A. fumigatus* , the MIC ranges (CLSI method) were as follows: amphotericin B 0.016–8 mg/L (37 studies), isavuconazole 0.06–32 mg/L (12 studies), itraconazole 0.015–128 mg/L (54 studies), posaconazole 0.008–32 mg/L (46 studies) and voriconazole 0.03–32 mg/L (62 studies); the corresponding ranges (EUCAST method) were as follows: amphotericin B 0.015–2 mg/L (eight studies), isavuconazole 0.06–32 mg/L (20 studies), itraconazole 0.015–128 mg/L (54 studies), posaconazole 0.008–32 mg/L (46 studies) and voriconazole 0.03–32 mg/L (62 studies), respectively (Table S7).

For *A. niger* , the MIC ranges (CLSI method) were as follows: amphotericin B 0.016–16 mg/L (31 studies), isavuconazole 0.06–16 mg/L (16 studies), itraconazole 0.015–16 mg/L (25 studies), posaconazole 0.002–4 mg/L (18 studies) and voriconazole 0.03–16 mg/L (28 studies); the corresponding ranges (EUCAST method) were as follows: amphotericin B 0.016–16 mg/L (31 studies), isavuconazole 0.06–16 mg/L (16 studies), itraconazole 0.06–16 mg/L (four studies), posaconazole 0.002–4 mg/L (23 studies) and voriconazole 0.25–2 mg/L (six studies), respectively (Table S7).

### 3.8 MIC Ranges of Antifungal Agents for Mucorales Isolates

For *Mucor* spp., the MIC ranges (CLSI method) were as follows: amphotericin B 0.03–8 mg/L (seven studies), isavuconazole 0.5–32 mg/L (six studies), itraconazole 0.03–32 mg/L (seven studies), posaconazole 0.008–8 mg/L (nine studies) and voriconazole 0.031–32 mg/L (nine studies), respectively (Table S8).

For *Rhizomucor* spp., the MIC ranges (CLSI method) were as follows: amphotericin B 0.03–1 mg/L (five studies), isavuconazole 0.06–8 mg/L (six studies), itraconazole 0.03–4 mg/L (five studies), posaconazole 0.03–4 mg/L (seven studies) and voriconazole 2–16 mg/L (seven studies), respectively (Table S8).

For *Rhizopus* spp., the MIC ranges (CLSI method) were as follows: amphotericin B 0.032–32 mg/L (nine studies), isavuconazole 0.125–32 mg/L (seven studies), itraconazole 0.031–32 mg/L (seven studies), posaconazole 0.008–32 mg/L (11 studies) and voriconazole 0.03–32 mg/L (13 studies), respectively (Table S8). The corresponding ranges (EUCAST method) were as follows: amphotericin B 0.25–16 mg/L (two studies), itraconazole 0.25–16 mg/L (two studies) and voriconazole 2–16 mg/L (two studies), respectively (Table S8).

### 3.9 Susceptibility Data Among Different Subgroups

#### 3.9.1 MIC50 of Antifungal Agents for Clinical Isolates

For *A. flavus* , the MIC50 values (CLSI method) were as follows: amphotericin B 2 (0.125–4) mg/L (19 studies), isavuconazole 1 (0.5–2) mg/L (12 studies), itraconazole 0.5 (0.125–16) mg/L (18 studies), posaconazole 0.25 (0.125–0.5) mg/L (19 studies) and voriconazole 0.5 (0.125–1) mg/L (19 studies); the corresponding values (EUCAST method) were as follows: amphotericin B 2.5 (1–32) mg/L (six studies), isavuconazole 1 (1) mg/L (two studies), itraconazole 0.25 (0.12–1) mg/L (three studies), posaconazole 0.125 (0.12–1) mg/L (one study) and voriconazole 0.5 (0.5–1) mg/L (seven studies), respectively (Figure S3A and Table S9).

For *A. fumigatus* , the MIC50 values (CLSI method) were as follows: amphotericin B 1 (0.0625–4) mg/L (24 studies), isavuconazole 0.5 (0.25–1) mg/L (14 studies), itraconazole 0.5 (0.125–1) mg/L (34 studies), posaconazole 0.25 (0.03–1) mg/L (31 studies) and voriconazole 0.5 (0.25–4) mg/L (38 studies); the corresponding values (EUCAST method) were as follows: amphotericin B 0.5 (0.25–3.12) mg/L (six studies), isavuconazole 1 (1) mg/L (three studies), itraconazole 0.5 (0.12–1) mg/L (two studies), posaconazole 0.125 (0.06–0.5) mg/L (five studies), and voriconazole 0.75 (0.5–4) mg/L (seven studies), respectively using (Figure S3B and Table S9).

For *A. niger* , the MIC50 values (CLSI method) were as follows: amphotericin B 0.5 (0.0312–8) mg/L (18 studies), isavuconazole 2 (1–2) mg/L (six studies), itraconazole 1 (0.032–4) mg/L (16 studies), posaconazole 0.5 (0.063–2) mg/L (11 studies), and voriconazole 1 (0.125–2) mg/L (18 studies); the corresponding values (EUCAST method) were as follows: amphotericin B 0.25 (0.12–6.25) mg/L (four studies), isavuconazole 1.25 (1–1.5) mg/L (two studies), itraconazole 0.5 (0.5–1) mg/L (two studies), posaconazole 0.125 (0.12–1) mg/L (three studies) and voriconazole 1 (0.5–1) mg/L (five studies) respectively (Figure S3C and Table S9).

For *Mucor* spp., the MIC50 values (CLSI method) were as follows: amphotericin B 0.375 (0.125–0.5) mg/L (four studies), isavuconazole 8 (4–8) mg/L (five studies), itraconazole 4 (0.25–4) mg/L (five studies), posaconazole 1 (0.25–2) mg/L (six studies) and voriconazole 16.25 (0.5–32) mg/L (five studies), respectively (Figure S4A and Table S10).

For *Rhizomucor* spp., the MIC50 values (CLSI method) were as follows: amphotericin B 0.375 (0.125–0.5) mg/L (four studies), isavuconazole 2 (2) mg/L (four studies), itraconazole 1 (0.5–1) mg/L (two studies) and posaconazole 0.5 (0.25–0.5) mg/L (four studies) respectively (Figure S4B and Table S10).

For *Rhizopus* spp., the MIC50 values (CLSI method) were as follows: amphotericin B 0.5 (0.25–1) mg/L (four studies), isavuconazole 1 (0.5–2) mg/L (five studies), itraconazole 1 (0.25–2) mg/L (five studies), posaconazole 0.5 (0.25–0.5) mg/L (six studies) and voriconazole 8 (0.5–8) mg/L (six studies) respectively (Figure S4C and Table S10).

#### 3.9.2 Susceptibility of Clinical Isolates to Antifungal Agents

For *A. flavus* , the susceptibility (CLSI method) was as follows: amphotericin B 98.55% (6.5%–100.0%) (eight studies), isavuconazole 97.9% (83.2%–100%) (two studies), itraconazole 100% (7.1%–100%) (nine studies), posaconazole 97.8% (57.9%–100%) (nine studies) and voriconazole 100% (95.8%–100%) (nine studies), respectively (Figure S5A and Table S11). The resistances to the drugs are provided in Figure S6A and Table S12.

For *A. fumigatus* , the susceptibility (CLSI method) was as follows: amphotericin B 99.85% (95.7%–100.0%) (11 studies), isavuconazole 95.1% (88.75%–96.2%) (five studies), itraconazole 93.35% (83.3%–100.0%) (14 studies), posaconazole 98% (79.4%–100.0%) (10 studies) and voriconazole 96.8% (91.1%–100.0%) (15 studies), respectively (Figure S5B and Table S11). The resistances are provided in Figure S6B and Table S12.

For *A. niger* , the susceptibility (CLSI method) was as follows: amphotericin B 100% (100.0%) (six studies), isavuconazole 97.75% (95.7%–100.0%) (three studies), itraconazole 96.9% (93.3%–100.0%) (seven studies), posaconazole 100% (95.5%–100.0%) (seven studies) and voriconazole 99.15% (76.2%–100%) (eight studies), respectively (Figure S5C and Table S11). The resistances are provided in Figure S6C and Table S12.

For *Mucor* spp., *Rhizomucor* spp. and *Rhizopus* spp., two studies reported resistances to amphotericin B, isavuconazole, itraconazole, posaconazole and voriconazole. The results were as described previously in ‘Susceptibility to antifungal agents used in treatment of mucormycosis’ (Table S13).

#### 3.9.3 MIC50 of Antifungal Agents for *Aspergillus* spp. Isolated From Environment

The MIC50 values of antifungal agents for *Aspergillus* spp. isolated from the environment are provided in Table S14. Susceptibility and resistance data for these isolates are provided in Tables S15 and S16, respectively. Analysis with a small number of studies on environmental isolates indicated that environmental strains were resistant to itraconazole or posaconazole, but clinical strains were still highly sensitive to itraconazole or posaconazole.

### 3.10 Susceptibility Data in Different Patient Populations

Susceptibility and resistance data in different patient populations with haematological diseases, invasive fungal infections, aspergillosis, otomycosis or otitis externa are provided in Table S17. The susceptibility data vary in different patient populations. The most clinical isolates were sensitive to the antifungal agents. However, the limited data indicated that *A. flavus* appeared to be resistant to amphotericin B or itraconazole.

## 4 Discussion

This review included 96 studies from multiple countries or regions. This study comprehensively summarised the MIC 50, susceptibility and resistance of *Aspergillu*s isolates and Mucorales isolates to antifungal agents, including amphotericin B, isavuconazole, itraconazole, posaconazole and voriconazole, for clinical and environmental *Aspergillu*s isolates and Mucorales isolates, which were the most common fungal pathogens in clinical practice [109] and a major threat to some patients, especially those with immunosuppression or immune deficiency [2]. With these data, we provided some detailed important information about antifungal treatment which could be referred to when necessary. MIC 50 is more commonly used and has been adopted by more studies compared with MIC 90. The choice of MIC 50 can greatly contribute to the estimation of pharmacodynamic in clinical application.

This study revealed that for certain *Aspergillus* isolates, the MIC 50 of some drugs was at or below the ECV, indicating that the isolates were WT strains [110], while the MIC 50 of other drugs exceeded the ECV and were elevated compared with existing MIC distributions. For example, for *A. flavus* and *A. niger* , the MIC values of voriconazole were below the ECV indicating these two fungi were sensitive to this drug, while for all three kinds of *Aspergillus* isolates, the MIC values of amphotericin B exceeded the ECV. However, the existing data was insufficient to compare the MIC 50 with ECV for Mucorales isolates. We believed that these data would be helpful for clinicians to choose reasonable antifungal drugs when facing fungal infections [111].

The EUCAST recently introduced an ATU as a warning to alert the laboratory to the uncertainty of the MIC result and that the laboratory needs to decide how to react to the warning before reporting a susceptibility classification to the clinician [108]. Normally, values between the S and R categories should be classified as I, but in the case of isolates with posaconazole MICs falling in 0.25 mg/L should not be interpreted as I but only as ATU. If S to itraconazole: report as S and add the following comment: The MIC is 0.25 mg/L and thus one dilution above the S breakpoint due to overlapping WT and non‐WT populations. If not S to itraconazole: report as R and refer to the reference laboratory for Cytochrome P450 proteins (CYP) 51A sequencing and confirmation of MICs [112].

Breakpoints can categorise an isolate as either susceptible or resistant, while the ECVs/ECOFFs can distinguish the WT (no known resistance mechanisms) from the non‐WT (harbouring resistant mechanisms). There has been a recent rise in non‐WT *Aspergillus* isolates, particularly in relation to azoles [113]. Drug resistance is a worrisome and widespread phenomenon that can result in treatment failure and a high fatality rate. A group of experts recommended that changes in treatment practices, including prolonging treatment time or multi‐drug combinations, should be used in areas where resistance is prevalent [114].

Breakpoints are used to predict whether an antifungal agent will be clinically effective against a particular fungal isolate. They are based on a combination of MIC distributions, ECV or ECOFFs, preclinical and clinical PK/PD, Monte Carlo simulations, and PK/PD breakpoints and clinical data, especially from a clinical trial. However, for many fungus–antifungal combinations, these data might never be available. For these combinations, epidemiological cutoff values can provide a methodology for categorising isolates either WT or non‐WT and provide some guidance for treatment [115]. According to the procedures for the development of ECV for antifungal susceptibility testing, for each ECV, data must be acquired from a minimum of three different laboratories, and the data from all three laboratories must meet the criteria for inclusion. Then, weighting is performed by converting each MIC within each dataset, such that the total in each dataset equals 100 (to be performed by the programme used to generate the ECVs). After weighing, the data can then be pooled. For each ECV, a minimum of 100 MIC/minimal effective concentration (MEC) values from 100 individual isolates are needed [107]. There is, to date, no international standard method for selecting ECV or ECOFFs. The definitions of ECOFF by EUCAST are similar but not identical to ECV, where the EUCAST approach is more prescriptive about the analysis. The setting of ECOFFs codifies its approach in the EUCAST standard operating procedure SOP 10.2 [116].

When there are no breakpoints or epidemiological cutoff values established for some antifungal agents, whether the MIC is within the WT population of that specific species or not can only be a reference for clinicians. We should identify the isolates and search relevant literature combing with the patient's clinical symptoms, other etiological results and therapeutic drugs to determine the clinical significance and importance of the species and adjust the medication regimen.

The summary also showed that the general susceptibility of *Aspergillus* isolates to antifungal agents using the CLSI method or EUCAST method was similar, and the resistance rate of *Aspergillus* isolates to amphotericin B was probably the lowest when compared with other drugs. However, we found no qualified data reporting drug sensitivity in Mucorales isolates. Limited data reported the resistance of Mucorales isolates to antifungal agents, suggesting that the resistance to amphotericin B was also the lowest.

Drug resistance is a major problem in clinical practice for patients with infection [6, 117]. As for fungi, the molecular mechanisms of drug resistance mainly involve (1) Target modification: fungi alter the target site to reduce the binding of drugs, (2) Target overexpression: fungi produce more of the binding‐receptor to dilute the effect of the drugs, (3) Efflux pumps: individuals actively pump out the antifungal agent to reduce intracellular concentration, (4) Biochemical target alteration: fungi produce alternative enzymes that can replace the target of the antifungal and (5) Metabolic inactivation: infected patients break down the antifungal agent through metabolic pathways [117, 118, 119, 120]. In the present study, we listed susceptibility and resistance according to both CLSI method and EUCAST method. These two methods have significant differences in their approaches and methodologies [121, 122]. CLSI uses ECVs to differentiate between wild‐type and non‐wild‐type strains, while EUCAST sets breakpoints that classify isolates as susceptible or resistant. The conditions for antifungal susceptibility testing, such as temperature and incubation times, are different between CLSI and EUCAST. Meanwhile, EUCAST breakpoints are often more stringent than CLSI's, leading to differences in susceptibility classification. Unlike CLSI, EUCAST has eliminated the intermediate category for some drugs, which might impact the agreement between the two [123, 124]. In our study, the data of susceptibility and resistance were slightly different between these two methods, especially in MIC values and MIC ranges. However, the results of variation trends in susceptibility and resistance and the identification of non‐WT and WT were basically consistent.

We also presented the MIC ranges of antifungal agents for *Aspergillus* isolates and Mucorales isolates in detail. From the results we listed, we can see that the ranges were wide with upper limit as hundreds or thousands of times of lower limit, indicating that even for the same kind of fungus, the MIC might be significantly different due to different studies, isolates, population and laboratory conditions. These results of MIC ranges provided references for clinicians and investigator to evaluate the performance of a specific antifungal agent.

Further, we summarised the MIC50 (CLSI method and EUCAST method) of antifungal agents for clinical isolates. The results showed that the two methods also resulted in similar MIC 50 values, which were consistent with those for whole isolates. We also summarised the susceptibility of clinical isolates (CLSI method) to antifungal agents, which was similar to general susceptibility described before. The results suggested that most clinical *Aspergillus* isolates of all 3 kinds were sensitive to the five antifungal agents, which was consistent with previous studies [96, 125, 126]. As for the resistance of clinical *Aspergillus* isolates to antifungal agents, voriconazole showed better performance, especially in *A. flavus* isolates. For clinical Mucorales isolates, there was also no study reporting the drug sensitivity. According to limited studies, clinical Mucorales isolates had lower resistance to amphotericin B when compared with other agents [127].

Aspergillosis or mucormycosis is an infectious disease for which person‐to‐person transmission seems unlikely. Patients likely become infected by inhaling strains present in the environment [128]. The environmental route probably plays an important role in the emergence of resistant strains [129]. The drug‐resistant mutations were first found in environmental strains and later found in clinical strains. Indeed, no previous azole treatment was reported for 50%–71% of aspergillosis cases caused by azole‐resistant strains [130, 131]. It is thus essential to pay attention to the screening of environmental resistant strains and the collecting of patients' personal contact history, occupation and trauma history.

We summarised the MIC50 of antifungal agents for *Aspergillus* isolated from environment and the susceptibility and resistance of these isolates to drugs. The results revealed that all three kinds of *Aspergillus* isolates, especially *A. niger* , had high susceptibility and lower resistance to voriconazole. This result might be important for choosing drugs for fungus‐infected patients when no clinical isolates were obtained. At last, we summarised the susceptibility and resistance data (MIC50, MIC range, susceptibility and resistance) of different strains to antifungal agents in different populations and found significant heterogeneity among different studies and population.

This study has the following strengths: firstly, the search strategy was developed by professional information specialists; we established a study selection process with strict inclusion and exclusion criteria, then we searched both electronic databases and the references of relevant systematic reviews; secondly, the study screening and data extraction were independently conducted by two researchers to minimise bias. The efficacy of antifungal agents in treating aspergillosis and mucormycosis patients and the resistance of the isolates were first analysed by a systematic review of the relevant field. Similarly to other studies, our systematic review also has some limitations. For example, no breakpoints or epidemiological cutoff values (ECVs or ECOFFs) have been established for any antifungal agent against Mucorales; therefore, emerging resistance and susceptibility to the agents cannot be evaluated from the descriptive data. Due to insufficient data, we failed to analyse the differences in the MIC and in vitro susceptibility of antifungal agents against *Aspergillus* spp. or Mucorales, and their resistances to the drugs, between environmental and clinical isolates. Moreover, the susceptibility and drug resistance data in different patient populations were insufficient for subgroup analyses. Overall, the target studies and results were heterogeneous, meaning that broad, summary conclusions could not be made.

This review increases understanding of the susceptibility and resistance of fungi to different antifungal agents, which is essential for the prompt and efficient treatment of patients with aspergillosis and mucormycosis and for improving the outcome of such infections. Some of our estimates might deserve special attention, particularly the evaluation of resistance of antifungal agents against *Aspergillus* spp. or Mucorales. Although we estimated a relatively low prevalence of resistance of fungi to different antifungal agents, the rates of deaths in which resistant fungi infection plays a role were so much greater in immunocompromised patients. Regarding *Aspergillus* spp., we estimated that *A. flavus* had consistently high in vitro susceptibility to voriconazole; however, *A. fumigatus* and *A. niger* had consistently high in vitro susceptibility to amphotericin B. For Mucorales, the resistance to amphotericin B was consistently at the lowest level. Accordingly, voriconazole or amphotericin B may be primarily considered to treat aspergillosis caused by *A. flavus, A. fumigatus* or *A. niger* and amphotericin B remains the most effective drug for the treatment of mucormycosis. Correct detection of resistant *Aspergillus* spp. or Mucorales isolates is, for obvious reasons, crucial to avoid inappropriate treatment in clinical practice. In the future, it will be imperative to bolster the development of novel diagnostic techniques to ensure early, precise and rapid detection of clinical drug‐resistant fungal strains. Further clinical breakpoints and epidemiological cutoff values must be established urgently for filamentous fungi, especially for the Mucorales and antifungal agents. Using cutoff values, we can compare and analyse the susceptibility data and the resistance of *Aspergillus* spp. or Mucorales to antifungal agents. Sufficient data from studies with larger sample sizes will be required to compare the susceptibility profiles of environmental and clinical isolates and to analyze their resistance to antifungal agents in different patient populations. Nevertheless, there remain numerous challenges regarding the management of invasive mycoses due to intrinsic and acquired resistant fungi given the limited number of drug classes available. Several new drug antifungal agents, also including two with new modes of action (fosmanogepix and olorofim), are in advanced clinical development and hold promise for improved outcomes for patients with drug‐resistant infection [132].

## 5 Conclusion

Descriptive data of MIC, susceptibility and resistance revealed that, compared with existing MIC distributions and breakpoints or ECVs established by CLSI or EUCAST, for *A. flavus* , the posaconazole and voriconazole MIC values were normal but amphotericin B, isavuconazole and itraconazole MIC values were elevated; for *A. fumigatus* , isavuconazole and itraconazole MIC values were normal but amphotericin B, posaconazole and voriconazole MIC values were elevated; and for *A. niger* , isavuconazole, itraconazole and voriconazole MIC values were normal but amphotericin B and posaconazole MIC values were elevated. In addition, *A. flavus* had consistently high in vitro susceptibility to voriconazole. *A. fumigatus* and *A. niger* had consistently high in vitro susceptibility to amphotericin B, which had low resistance to the drug.

## Author Contributions

**Yinggai Song:** conceptualization, methodology, formal analysis, writing – review and editing. **Paul E. Verweij:** conceptualization, methodology, formal analysis, writing – review and editing. **Jochem B. Buil:** data curation, writing – review and editing, formal analysis. **Sybren de Hoog:** data curation, writing – review and editing, formal analysis. **Jie Liu:** data curation, conceptualization, methodology, writing – review and editing, formal analysis, project administration. **Jiaxian Guo:** data curation, formal analysis, conceptualization, methodology, writing – review and editing, project administration. **Wei Liu:** conceptualization, methodology, writing – review and editing, formal analysis, supervision. **Ruoyu Li:** conceptualization, methodology, writing – review and editing, supervision, formal analysis.

## Consent

The authors have nothing to report.

## Conflicts of Interest

Although Jie Liu and Jiaxian Guo were employees of MSD, all of the authors declare no conflicts of interest.

## Supporting information


**Data S1:** myc70118‐sup‐0001‐Supinfo1.docx.

## Data Availability

The original contributions presented in this study are included in the article/Supporting Information; further inquiries can be directed to the corresponding author.
