# Supplementary material for: Analysis of Susceptibility and Drug Resistance of Antifungal Agents in Aspergillosis and Mucormycosis Patients: A Systematic Review
Source: Mycoses. 2025 Oct 18;68(10):e70118. doi: 10.1111/myc.70118 (PMC12535282; doi:10.1111/myc.70118)
Supplement: Supplementary file 1 — Data S1: myc70118‐sup‐0001‐Supinfo1.docx. [file MYC-68-e70118-s001.docx]

Content

[Appendix 1 Search strategy 2](#_Toc198199580)

[Appendix Figure 1. Susceptibility of *Aspergillus* isolates to antifungal agents 7](#_Toc198199581)

[Appendix Figure 2. Resistance of *Aspergillus* isolates to antifungal agents 10](#_Toc198199582)

[Appendix Figure 3. MIC50 of antifungal agents for *Aspergillus* isolated from patients 13](#_Toc198199583)

[Appendix Figure 4. MIC50 of antifungal agents for Mucorales isolated from patients 16](#_Toc198199584)

[Appendix Figure 5. Susceptibility of *Aspergillus* isolated from patients to antifungal agents 19](#_Toc198199585)

[Appendix Figure 6. Resistance of *Aspergillus* isolated from patients to antifungal agents 22](#_Toc198199586)

[Appendix Table 1. MIC50 of antifungal agents for *Aspergillus* isolates 23](#_Toc198199587)

[Appendix Table 2. MIC50 of antifungal agents for Mucorales isolates 25](#_Toc198199588)

[Appendix Table 3. Susceptibility of Aspergillus isolates to antifungal agents 27](#_Toc198199589)

[Appendix Table 4. Resistance of *Aspergillus* isolates to antifungal agents 29](#_Toc198199590)

[Appendix Table 5. Resistance of Mucorales isolates to antifungal agents 31](#_Toc198199591)

[Appendix Table 6. MIC range of antifungal agents for *Aspergillus* isolates 33](#_Toc198199592)

[Appendix Table 7. MIC range of antifungal agents for Mucorales isolates 35](#_Toc198199593)

[Appendix Table 8. MIC50 of antifungal agents for Aspergillus isolated from patients 37](#_Toc198199594)

[Appendix Table 9. MIC50 of antifungal agents for Mucorales isolated from patients 39](#_Toc198199595)

[Appendix Table 10. Susceptibility of Aspergillus isolated from patients to antifungal agents 40](#_Toc198199596)

[Appendix Table 11. Resistance of *Aspergillus* isolated from patients to antifungal agents 42](#_Toc198199597)

[Appendix Table 12. Resistance of Mucorales isolated from patients to antifungal agents 44](#_Toc198199598)

[Appendix Table 13. MIC50 of antifungal agents for *Aspergillus* isolated from environment 45](#_Toc198199599)

[Appendix Table 14. Susceptibility of *Aspergillus* isolated from environment to antifungal agents 46](#_Toc198199600)

[Appendix Table 15. Resistance of *Aspergillus* isolated from environment to antifungal agents 47](#_Toc198199601)

[Appendix Table 16. Antifungal susceptibility and resistance data (MIC50, MIC range, susceptibility, and resistance) of different strains to antifungal agents in different populations 49](#_Toc198199602)

## Appendix 1 Search strategy

**PubMed**

1. "Aspergillus fumigatus"[Mesh] OR "aspergillus flavus"[Mesh] OR "aspergillus niger"[Mesh] OR "rhizopus"[Mesh] OR "rhizomucor"[Mesh] OR "mucor"[Mesh] OR "Aspergillus fumigatus"[tw] OR "aspergillus flavus"[tw] OR "aspergillus niger"[tw] OR "rhizopus"[tw] OR "rhizomucor"[tw] OR "mucor"[tw] OR "A. fumigatus"[tw] OR "a. flavus"[tw] OR "a. niger"[tw]
2. "isavuconazole" [Supplementary Concept] OR isavuconazole OR "BAL-8557" OR isavuconazonium OR Cresemba OR "posaconazole" [Supplementary Concept] OR posaconazole OR Noxafil OR "SCH-56592" OR "Amphotericin B"[Mesh] OR Amphotericin OR Fungizone OR Amphocil OR "Itraconazole"[Mesh] OR itraconazole OR canadiol OR candistat OR canditral OR hongoseril OR hyphanox OR itrac OR itralek OR itranax OR pulmazole OR "pur 1900" OR sporacid OR sporal OR sporonox OR triasporin OR R51211 OR Sporanox OR Orungal OR "Voriconazole"[Mesh] OR voriconazole OR "UK 109496" OR Vfend OR "zp 059"
3. "disease susceptibility"[Mesh] OR susceptibl*[tiab] OR susceptiv*[tiab] OR resist*[tiab] OR "hypersensitivity"[Mesh] OR hypersensitivit*[tiab] OR sensitiv*[tiab] OR "sensitivity and specificity"[Mesh] OR specificit*[tiab] OR activit*[tiab]
4. #1 and #2 and #3
5. #4 NOT ("Review" [Publication Type] OR "Review Literature as Topic"[Mesh] OR Review[ti])
6. #5 and ("2010/01/01"[Date - Publication] : "3000"[Date - Publication])
7. "in vitro"[tiab] OR isolat*[tiab]
8. #6 AND #7

**EMBASE**

1. 'Aspergillus fumigatus'/exp OR 'Aspergillus flavus'/exp OR 'Aspergillus niger'/exp OR 'Rhizopus'/exp OR 'Rhizomucor'/exp OR 'Mucor'/exp OR ("Aspergillus fumigatus" OR "aspergillus flavus" OR "aspergillus niger" OR "rhizopus" OR "rhizomucor" OR "mucor" OR "A. fumigatus" OR "a. flavus" OR "a. niger"):ab,ti,kw
2. 'isavuconazole'/exp OR 'posaconazole'/exp OR 'amphotericin B'/exp OR 'itraconazole'/exp OR 'voriconazole'/exp OR (isavuconazole OR "BAL-8557" OR isavuconazonium OR Cresemba OR posaconazole OR Noxafil OR "SCH-56592" OR Amphotericin OR Fungizone OR Amphocil OR itraconazole OR canadiol OR candistat OR canditral OR hongoseril OR hyphanox OR itrac OR itralek OR itranax OR pulmazole OR "pur 1900" OR sporacid OR sporal OR sporonox OR triasporin OR R51211 OR Sporanox OR Orungal OR voriconazole OR "UK 109496" OR Vfend OR "zp 059"):ab,ti,kw
3. 'sensitivity and specificity'/exp OR (susceptibl* OR susceptiv* OR resist* OR hypersensitivit* OR sensitiv* OR specificit* OR activit*):ab,ti
4. #1 and #2 and #3
5. #4 not ([review]/lim OR 'review'/exp OR review:ti)
6. #5 AND [2010-2022]/py
7. ("in vitro" or isolat*):ti,ab
8. #6 and #7

**Cochrane**

#1 MeSH descriptor: [Aspergillus fumigatus] explode all trees

#2 MeSH descriptor: [Aspergillus flavus] explode all trees

#3 MeSH descriptor: [Aspergillus niger] explode all trees

#4 MeSH descriptor: [Rhizopus] explode all trees

#5 MeSH descriptor: [Rhizomucor] explode all trees

#6 MeSH descriptor: [Mucor] explode all trees

#7 "Aspergillus fumigatus" OR "aspergillus flavus" OR "aspergillus niger" OR "rhizopus" OR "rhizomucor" OR "mucor" OR "A. fumigatus" OR "a. flavus" OR "a. niger"

#8 #1 or #2 or #3 or #4 or #5 or #6 or #7

#9 MeSH descriptor: [Amphotericin B] explode all trees

#10 MeSH descriptor: [Itraconazole] explode all trees

#11 MeSH descriptor: [Voriconazole] explode all trees

#12 (isavuconazole OR "BAL-8557" OR isavuconazonium OR Cresemba OR posaconazole OR Noxafil OR "SCH-56592" OR Amphotericin OR Fungizone OR Amphocil OR itraconazole OR canadiol OR candistat OR canditral OR hongoseril OR hyphanox OR itrac OR itralek OR itranax OR pulmazole OR "pur 1900" OR sporacid OR sporal OR sporonox OR triasporin OR R51211 OR Sporanox OR Orungal OR voriconazole OR "UK 109496" OR Vfend OR "zp 059"):ti,ab,kw

#13 #9 or #10 or #11 or #12

#14 MeSH descriptor: [Disease Susceptibility] explode all trees

#15 MeSH descriptor: [Hypersensitivity] explode all trees

#16 MeSH descriptor: [Sensitivity and Specificity] explode all trees

#17 (susceptibl* OR susceptiv* OR resist* OR hypersensitivit* OR sensitiv* OR specificit* OR activit*):ti

#18 (susceptibl* OR susceptiv* OR resist* OR hypersensitivit* OR sensitiv* OR specificit* OR activit*):ab

#19 #14 or #15 or #16 or #17 or #18

#20 ("in vitro" or isolat*):ti,ab,kw

#21 #8 and #13 and #19 and #20

|  | CLSI | EUCAST |
| --- | --- | --- |
| A. *A.* *flavus* | 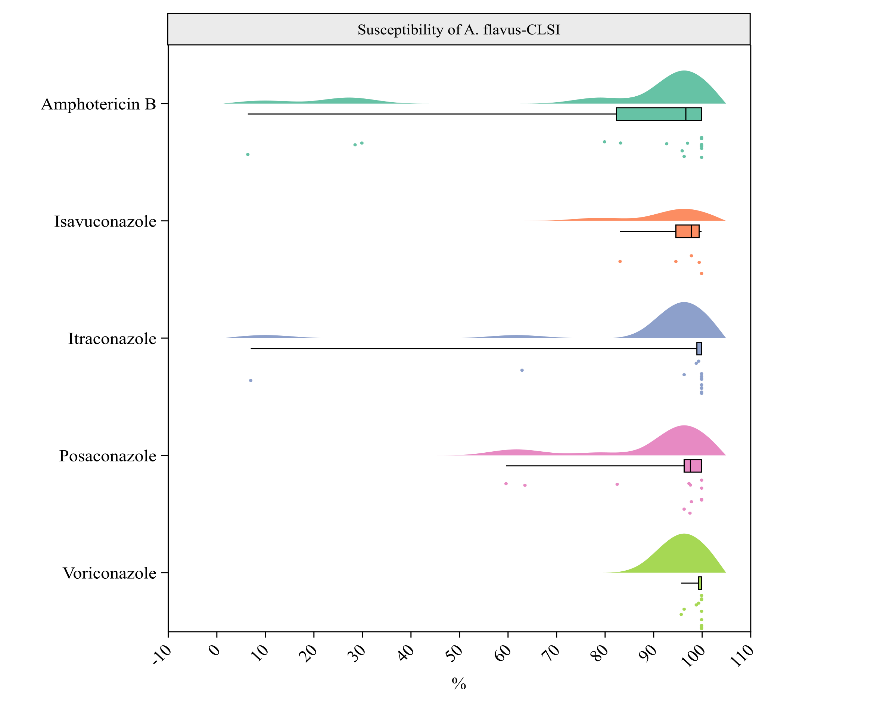 | 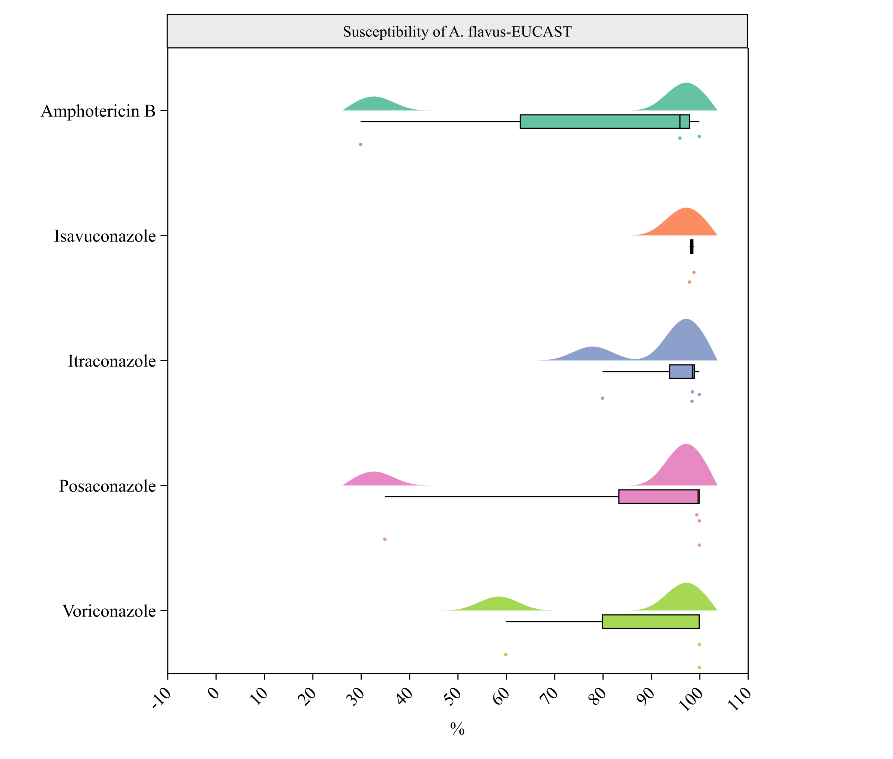 |
| B. *A.* *fumigatus* | 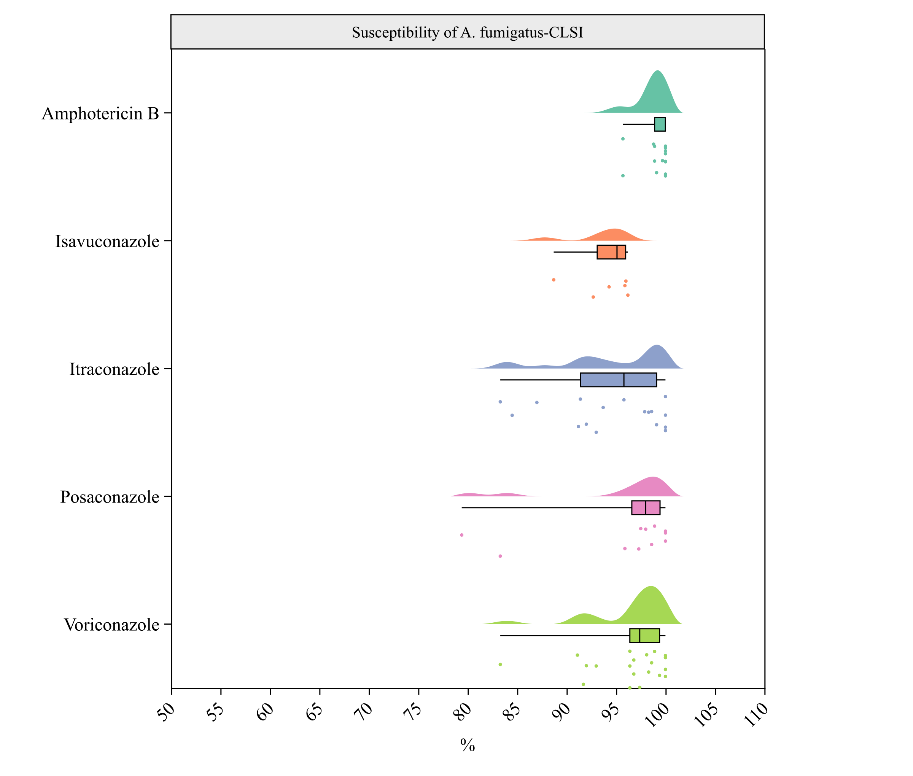 | 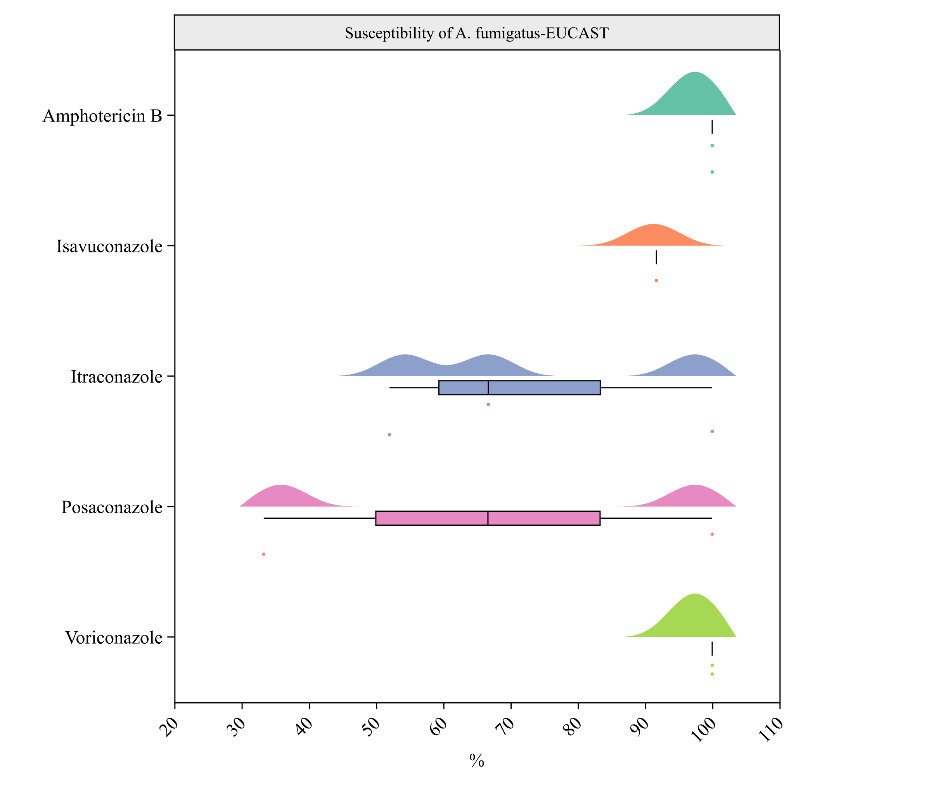 |
| C. *A.* *niger* | 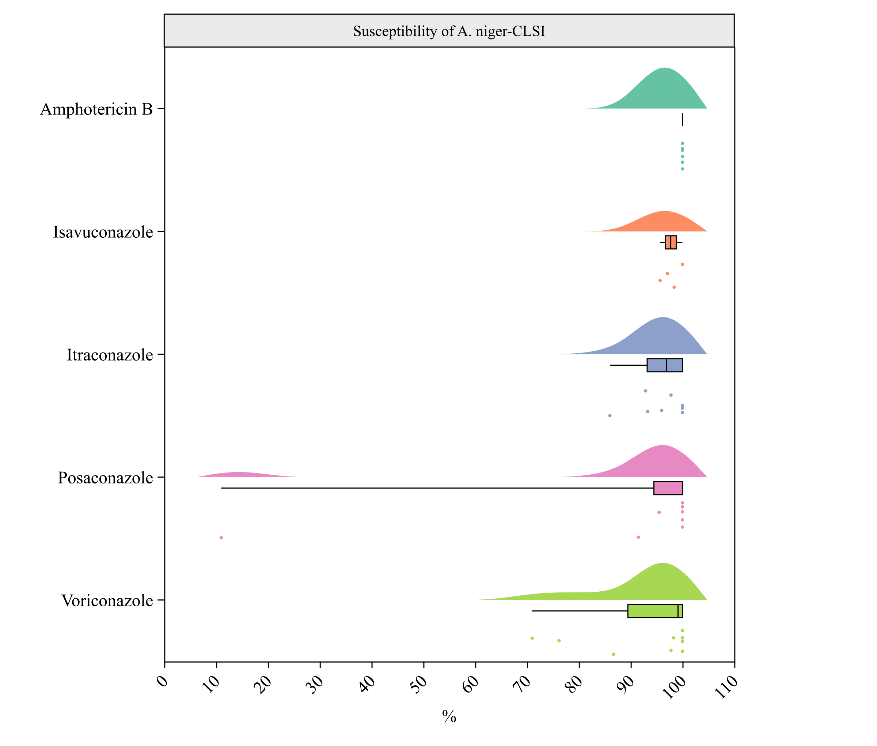 | 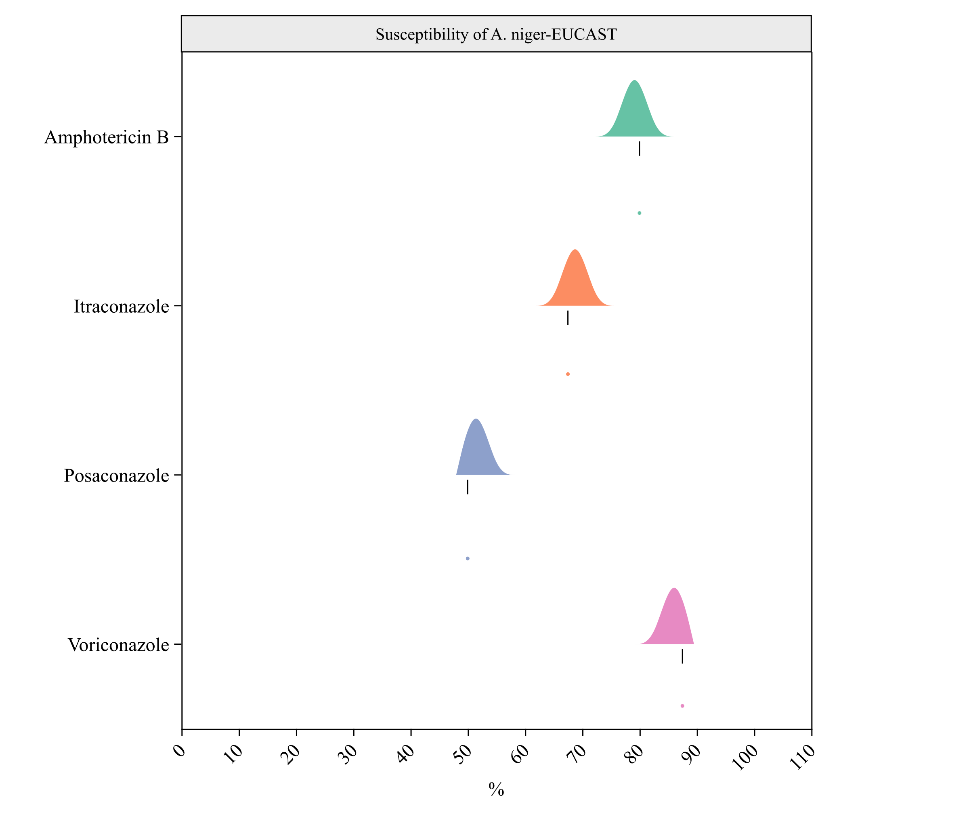 |

## Appendix Figure 1. Susceptibility of *Aspergillus* isolates to antifungal agents

|  | CLSI | EUCAST | | |
| --- | --- | --- | --- | --- |
| A. *A.* *flavus* | 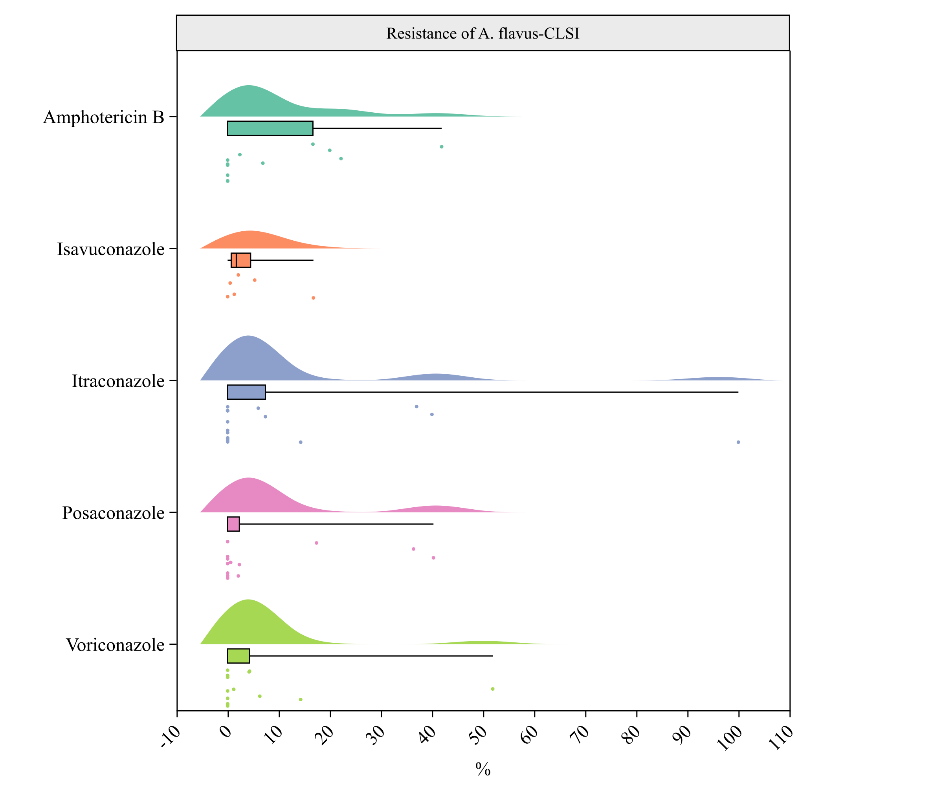 | 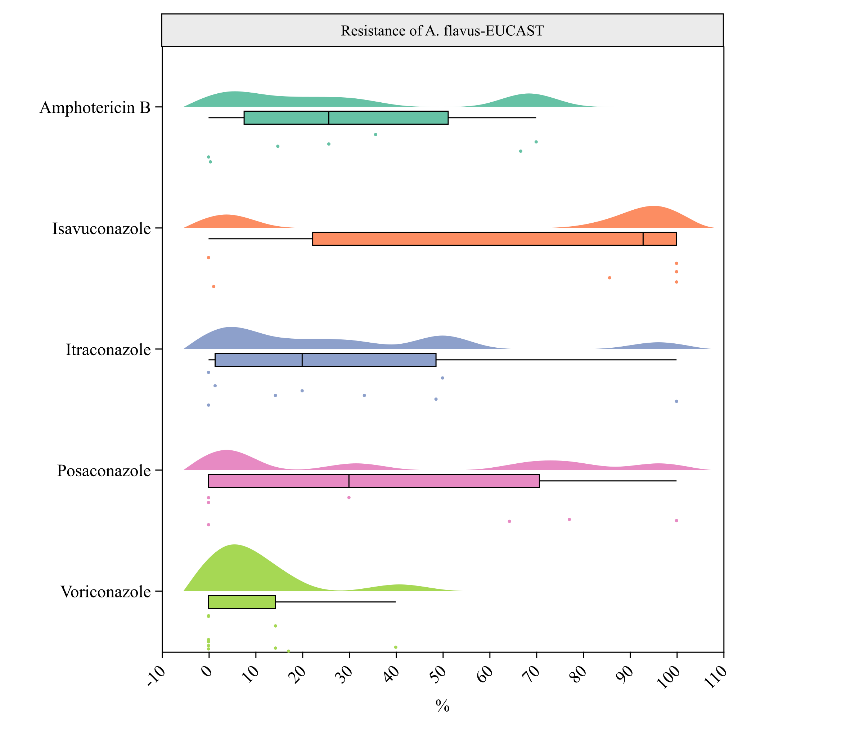 | | |
| B. *A.* *fumigatus* | 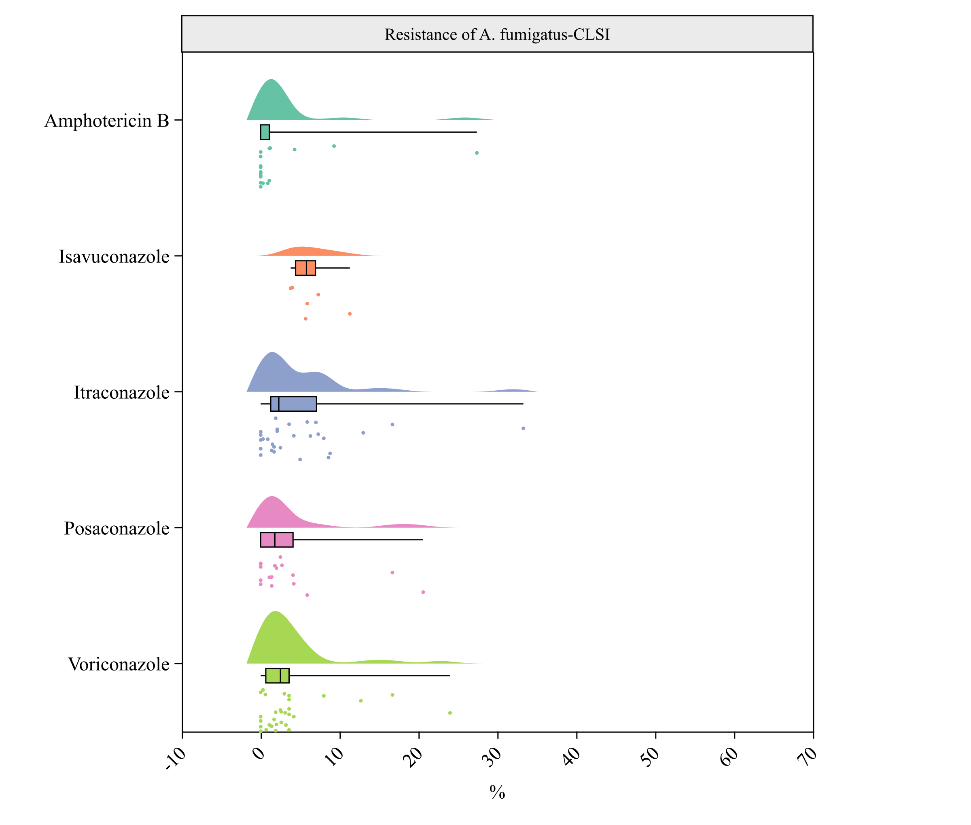 | | 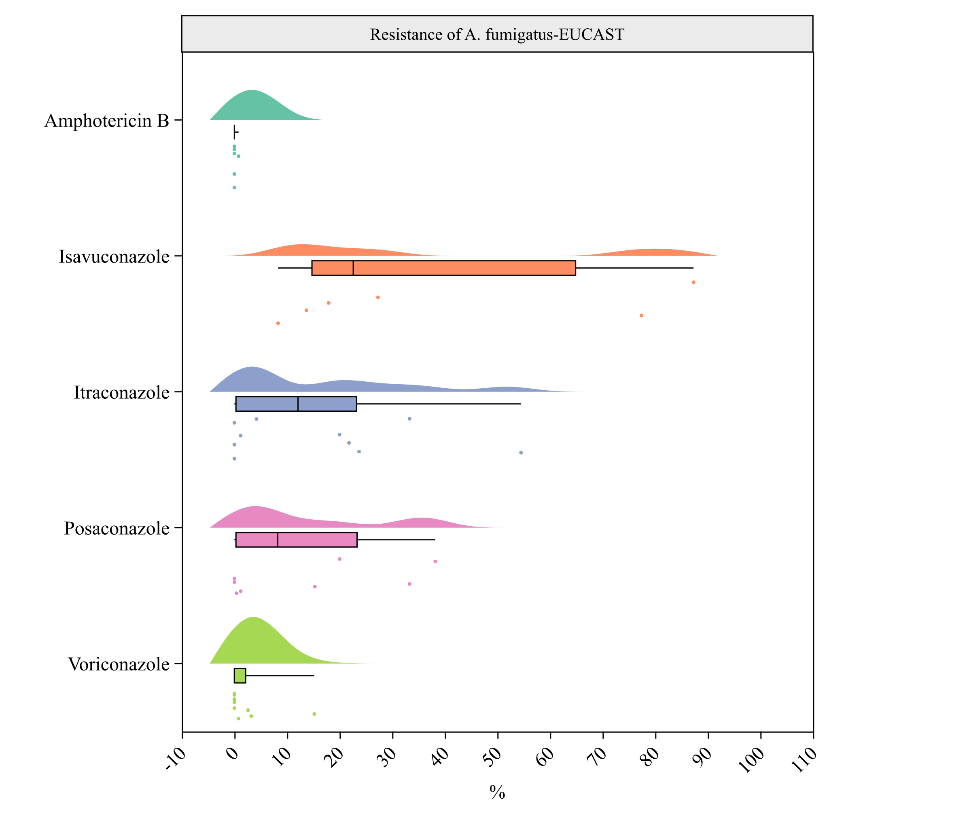 | |
| C. *A.* *niger* | 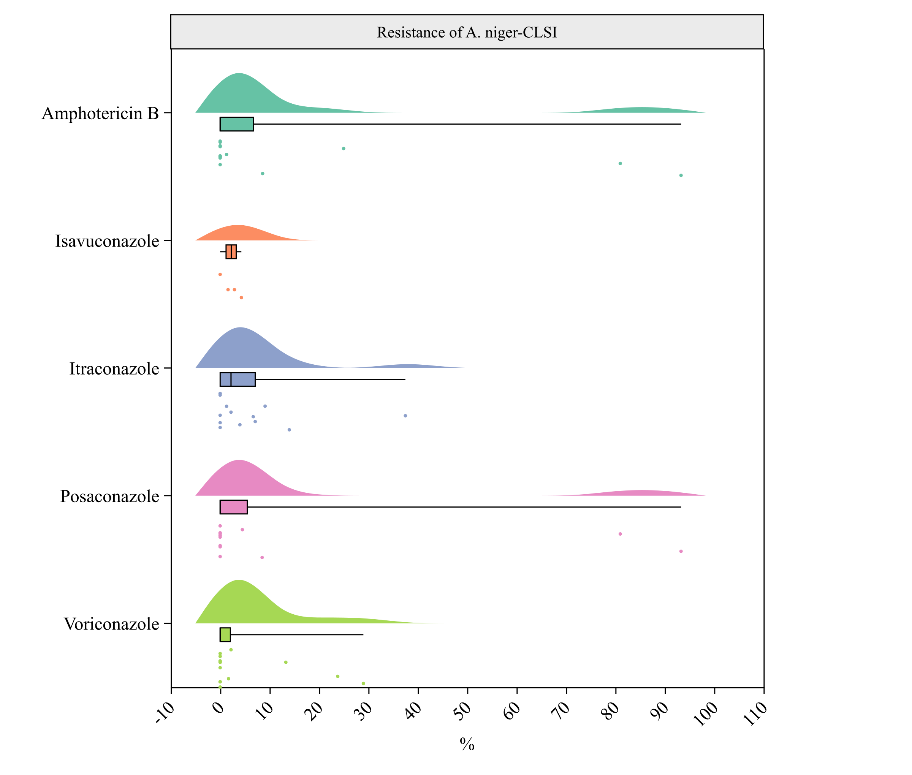 | | | 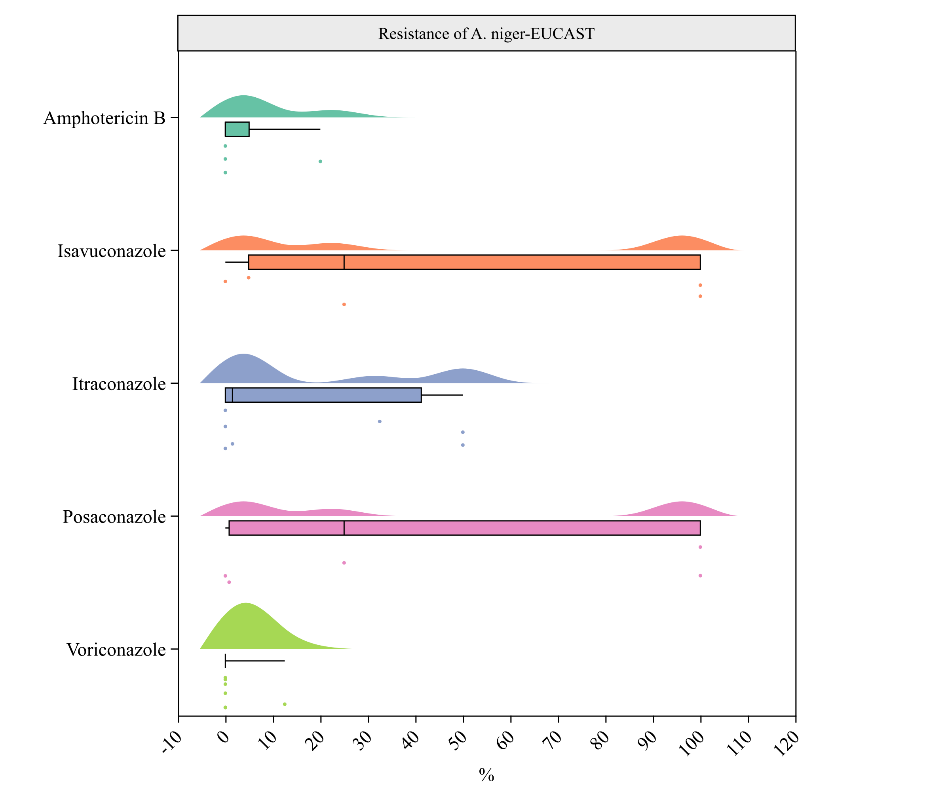 |

## Appendix Figure 2. Resistance of *Aspergillus* isolates to antifungal agents

|  | CLSI | EUCAST |
| --- | --- | --- |
| A. *A.* *flavus* | 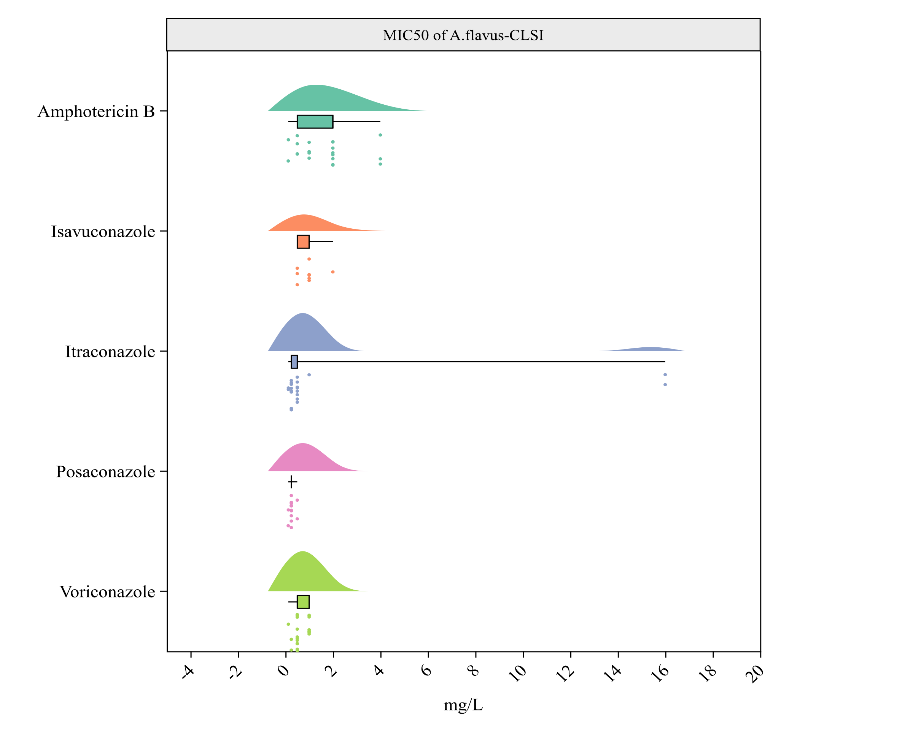 | 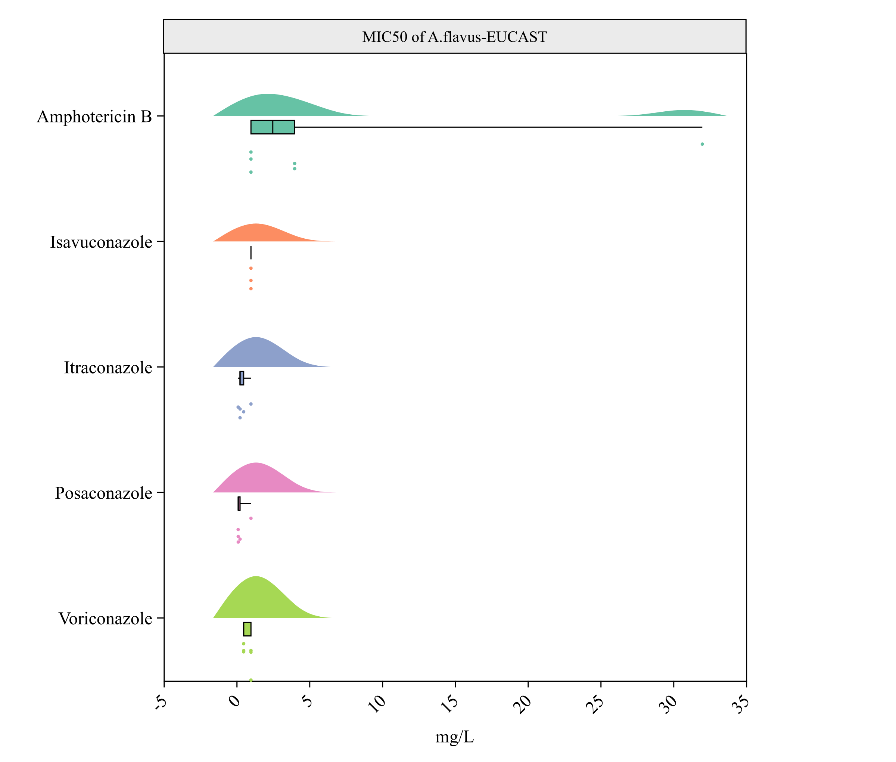 |
| B. *A.* *fumigatus* | 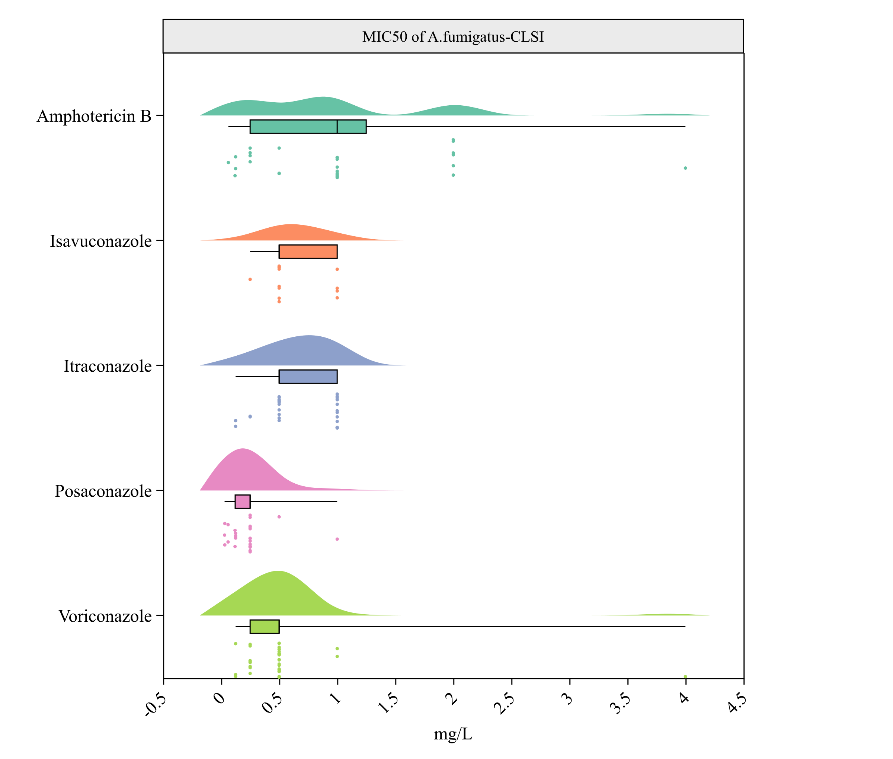 | 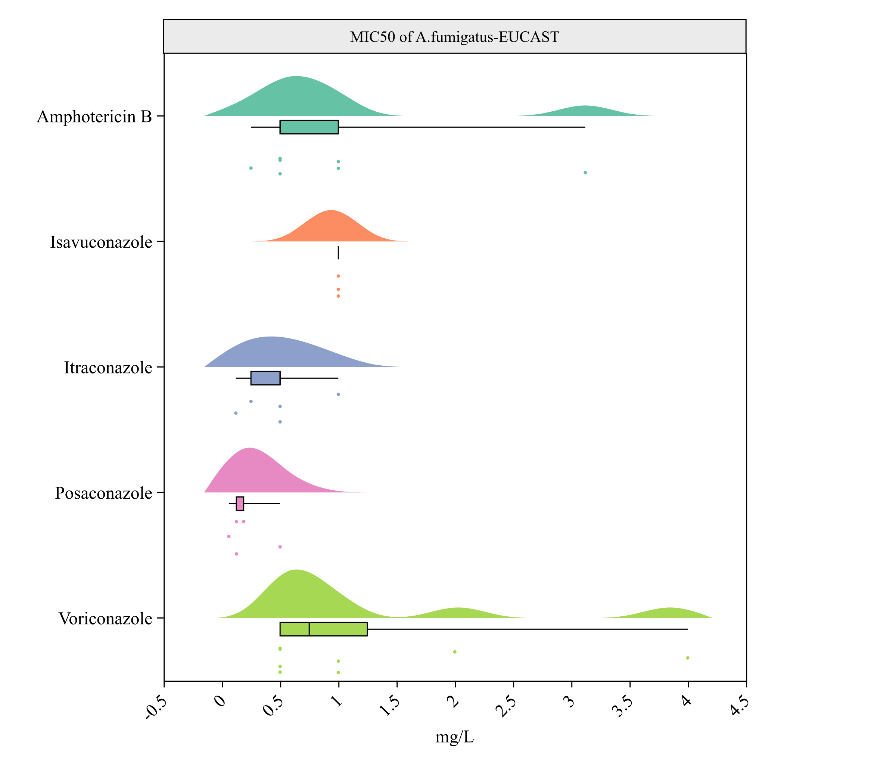 |
| C. *A.* *niger* | 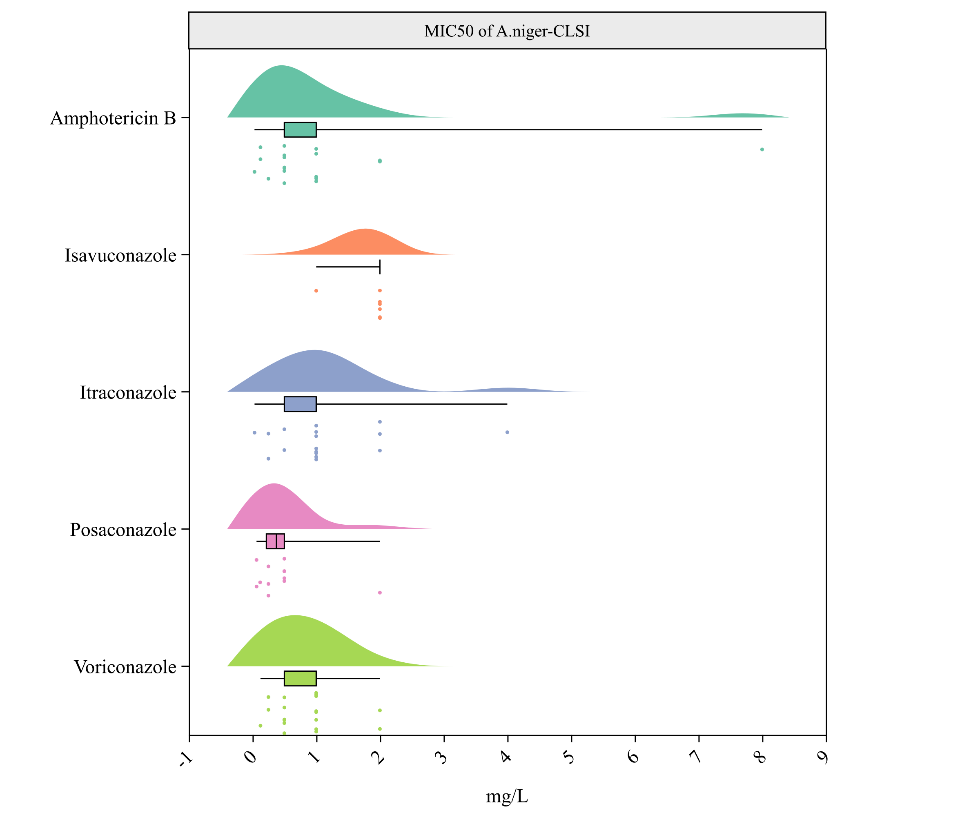 | 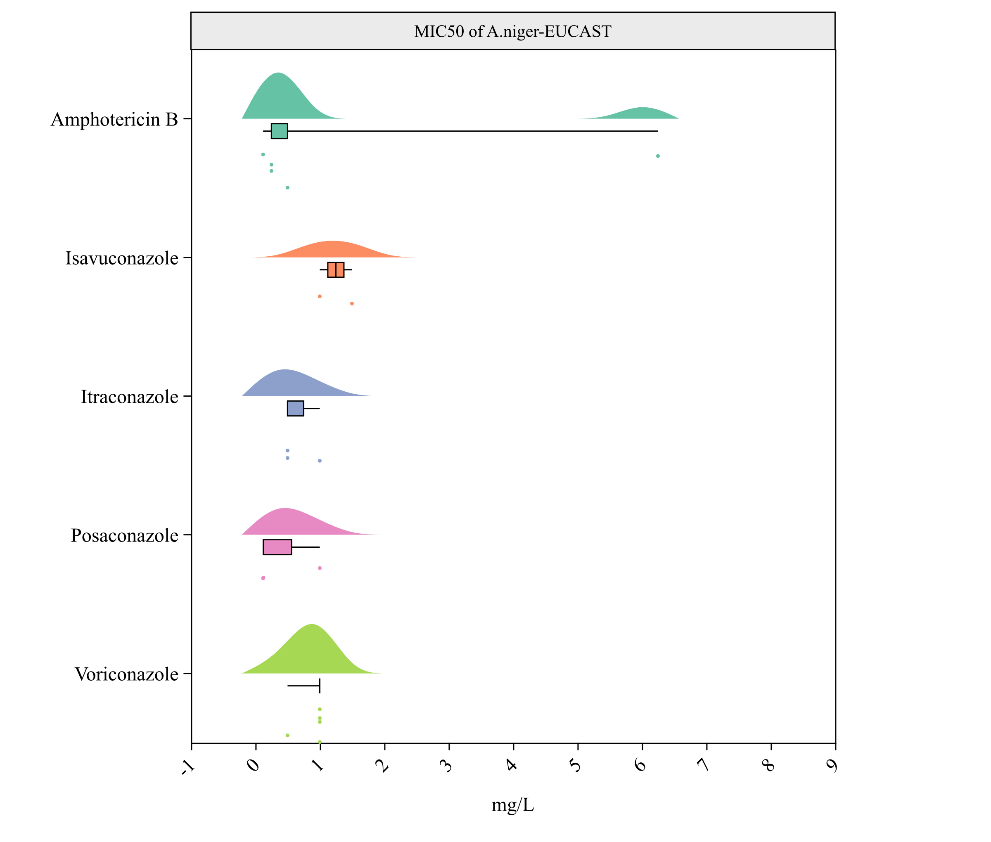 |

## Appendix Figure 3. MIC50 of antifungal agents for *Aspergillus* isolated from patients

|  | CLSI |
| --- | --- |
| A. *Mucor* spp. | 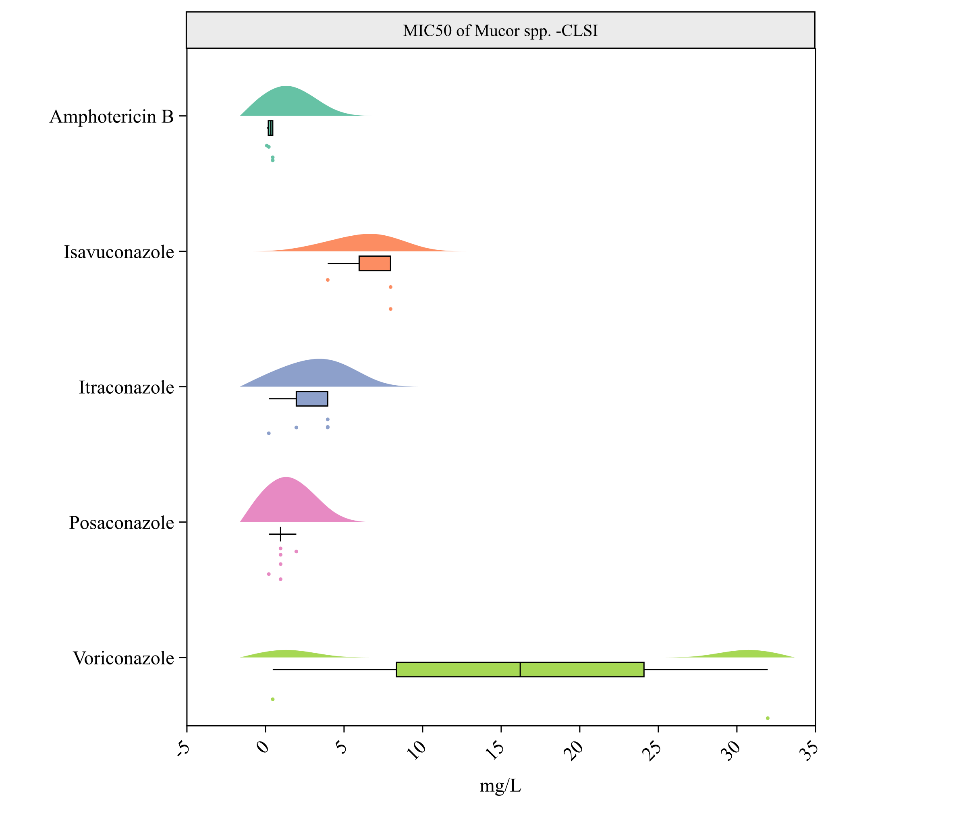 |
| B. *Rhizomucor* spp. | 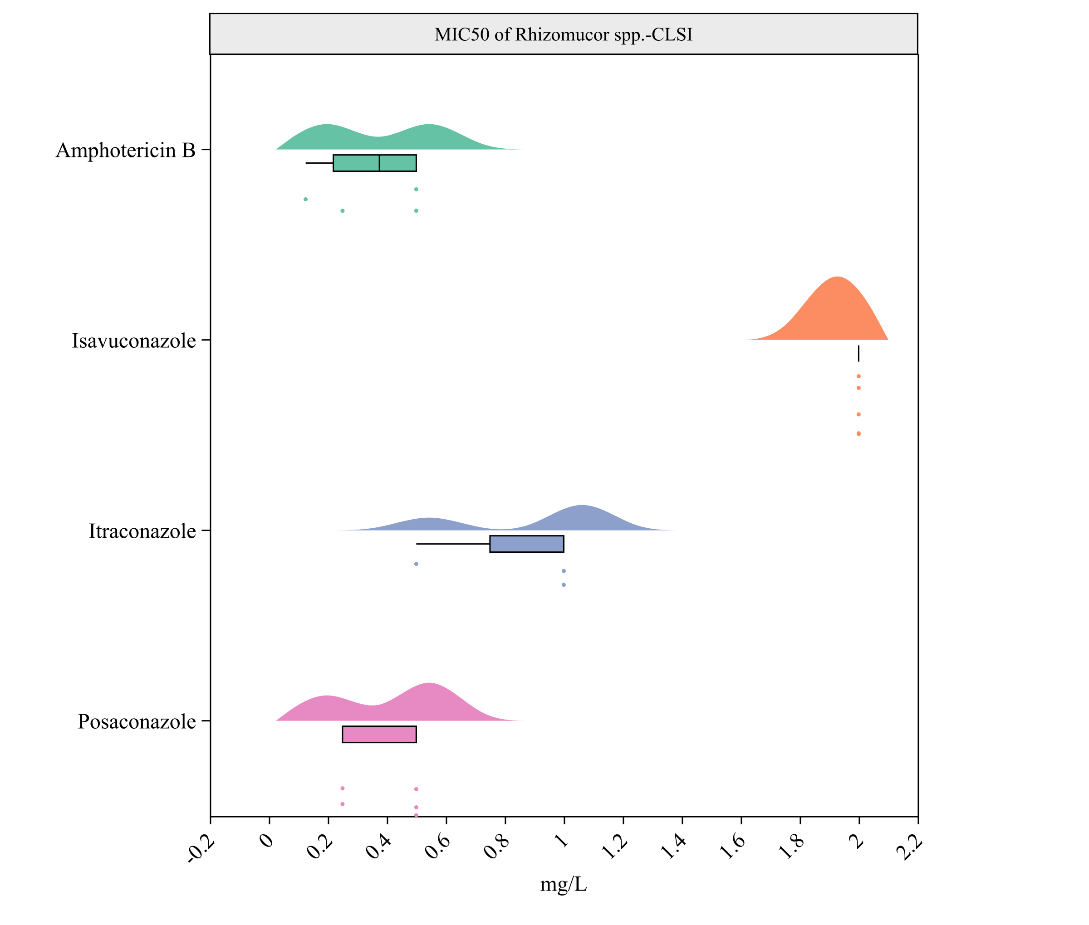 |
| C. *Rhizopus* spp. | 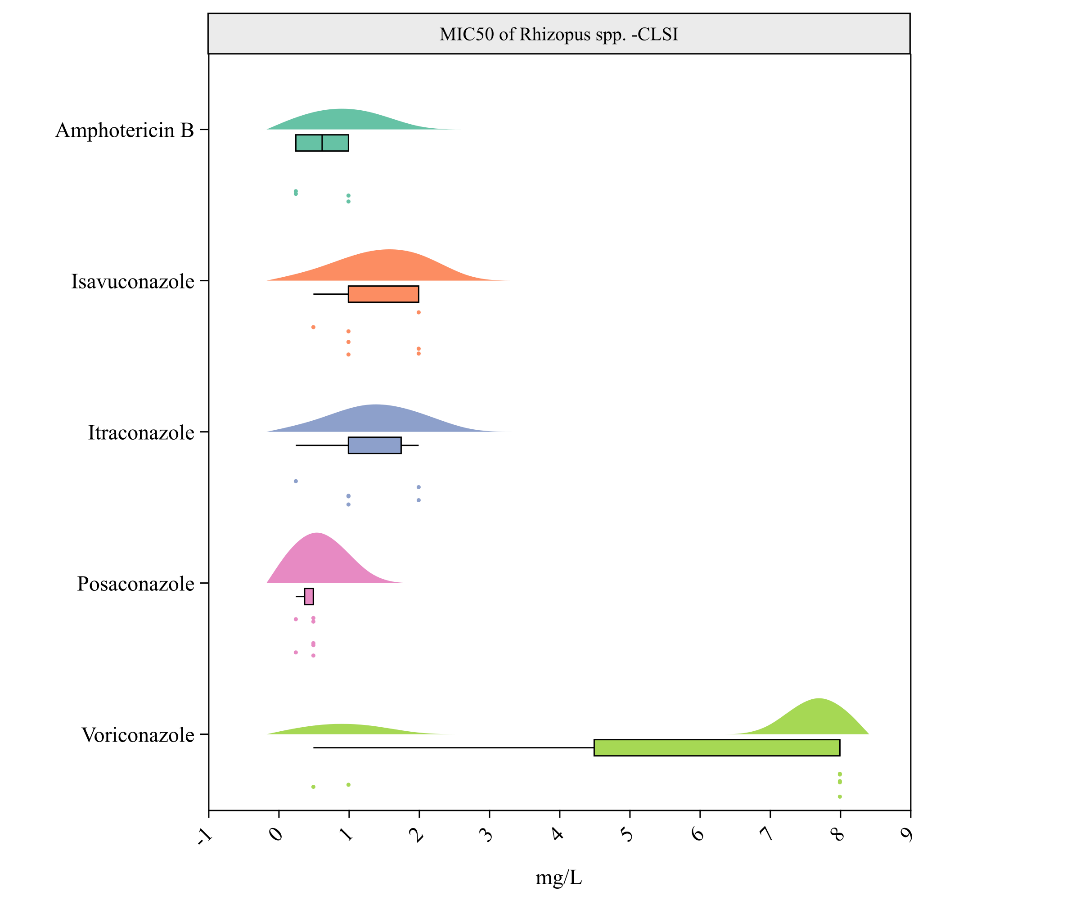 |

## Appendix Figure 4. MIC50 of antifungal agents for Mucorales isolated from patients

|  | CLSI | EUCAST |
| --- | --- | --- |
| A. *A.* *flavus* | 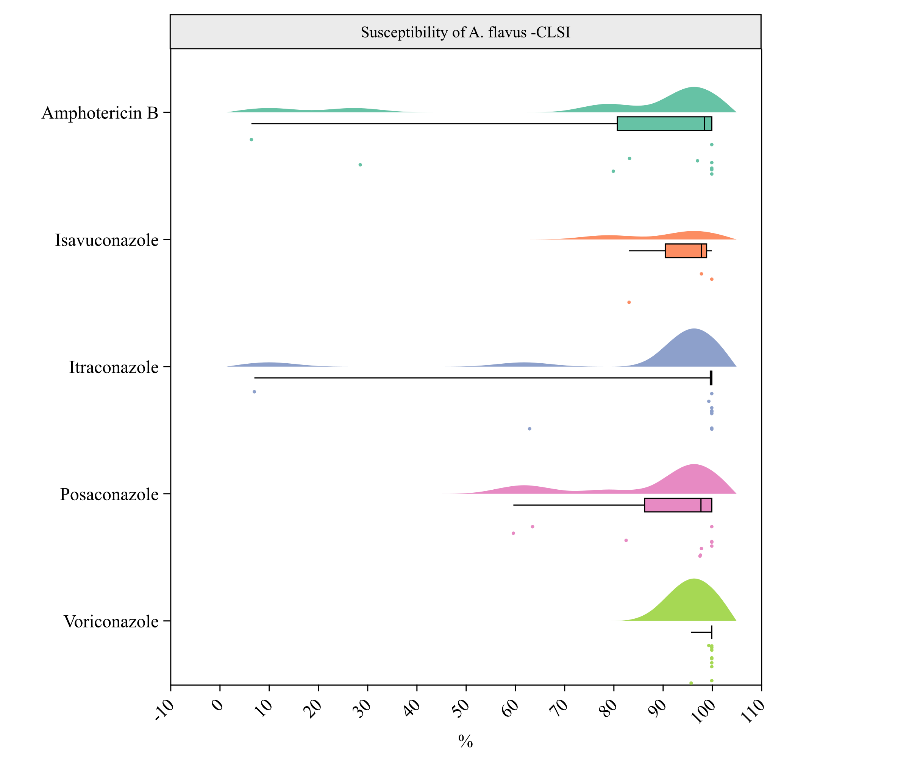 | 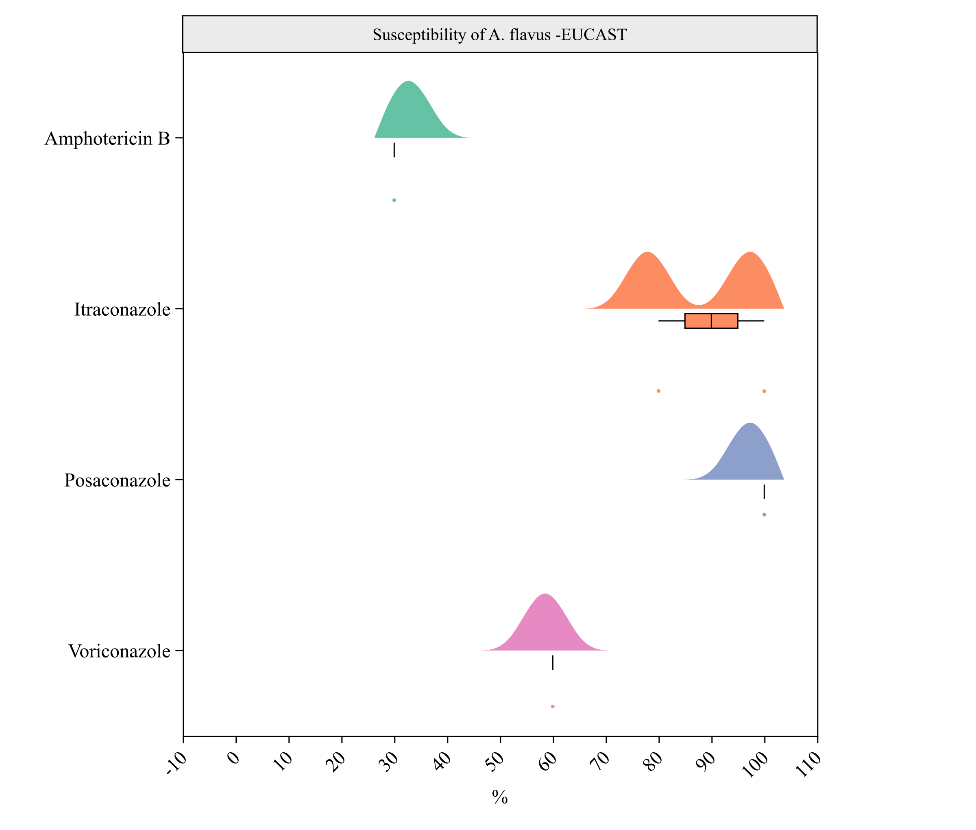 |
| B. *A.* *fumigatus* | 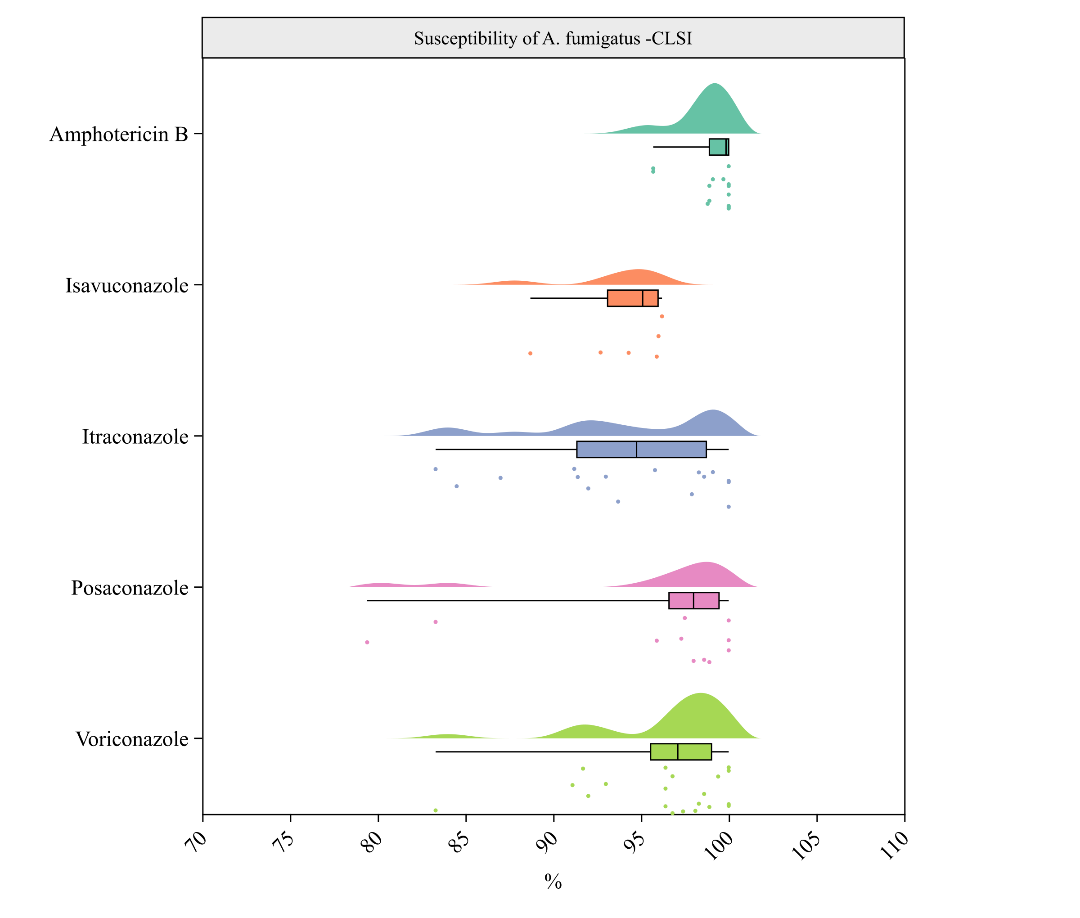 | 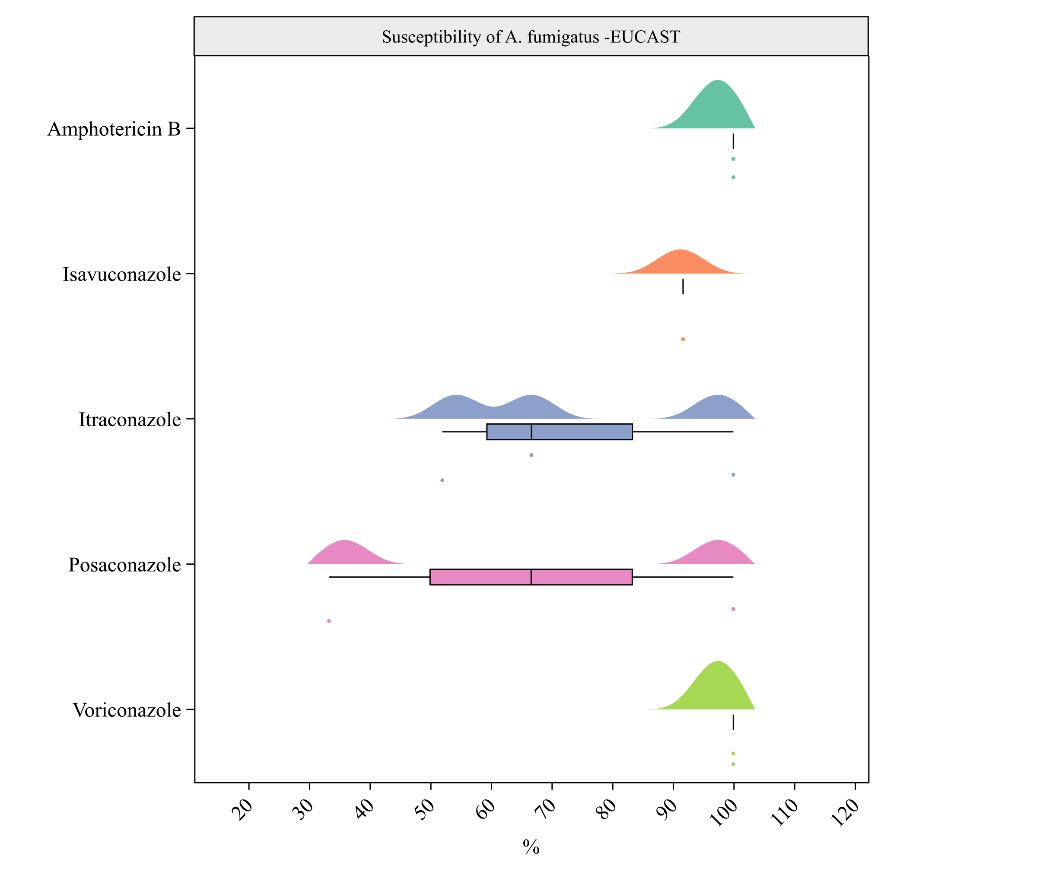 |
| C. *A.* *niger* | 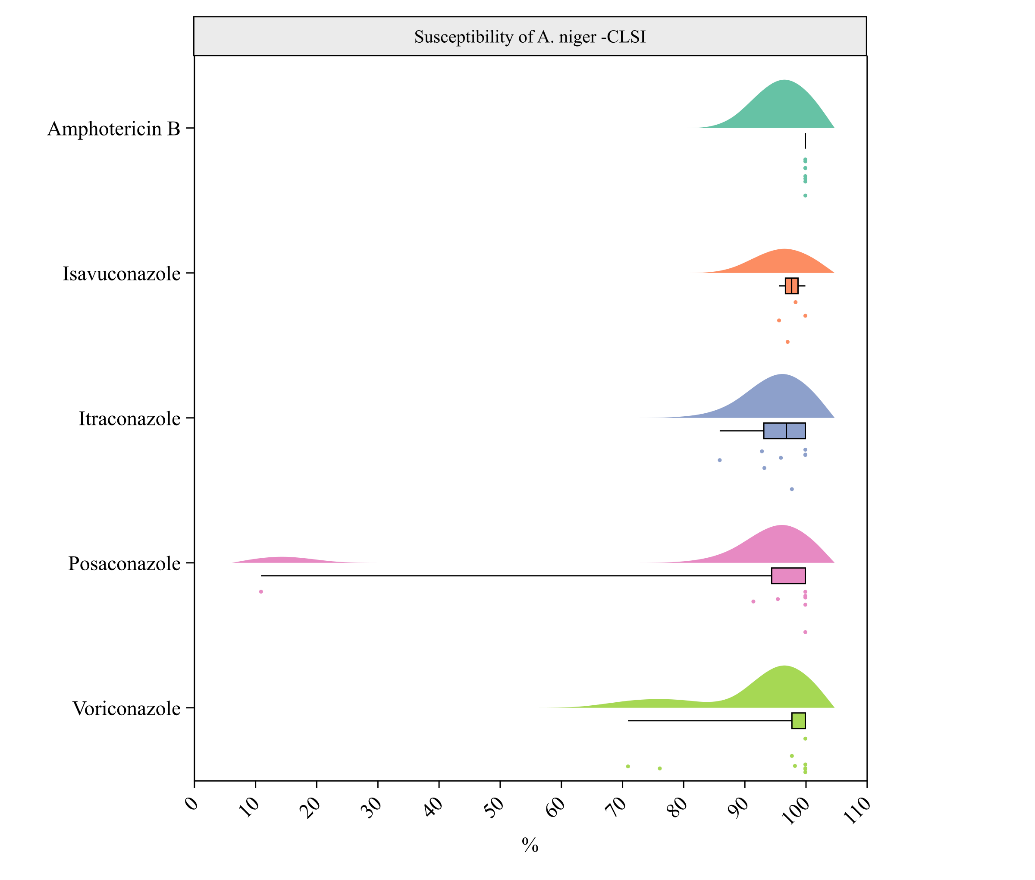 | 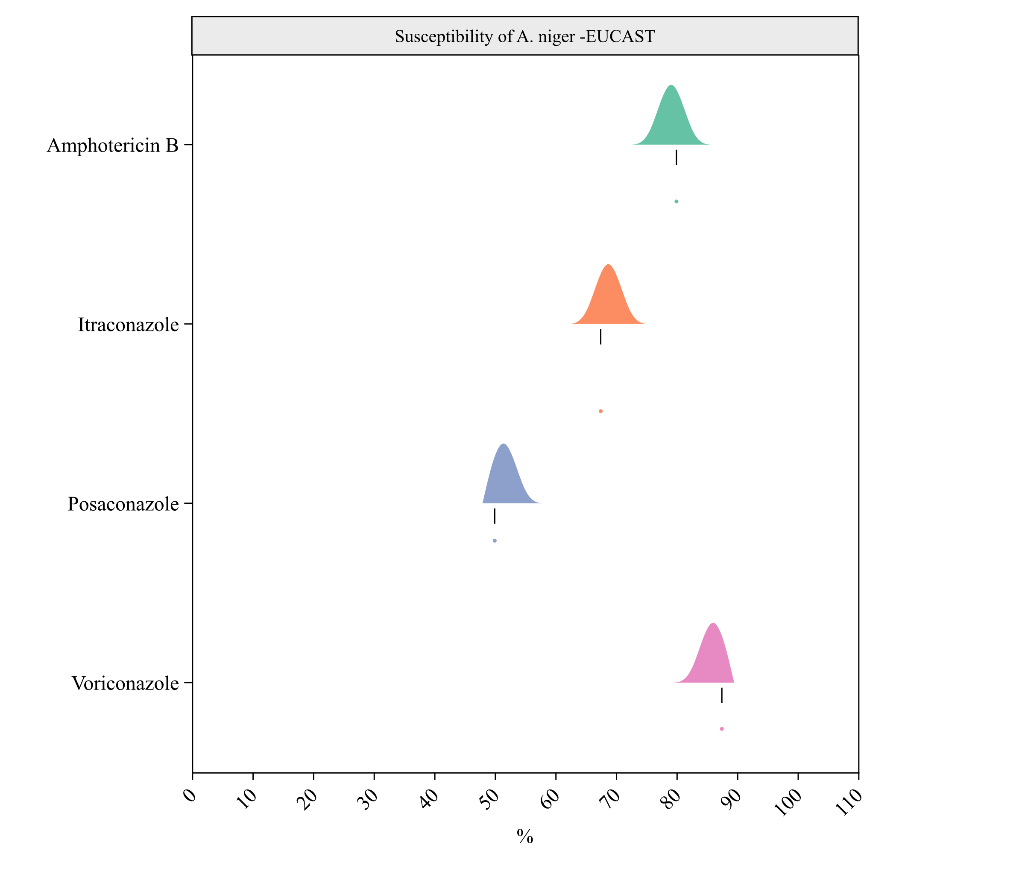 |

## Appendix Figure 5. Susceptibility of *Aspergillus* isolated from patients to antifungal agents

|  | CLSI | EUCAST |
| --- | --- | --- |
| A. *A.* *flavus* | 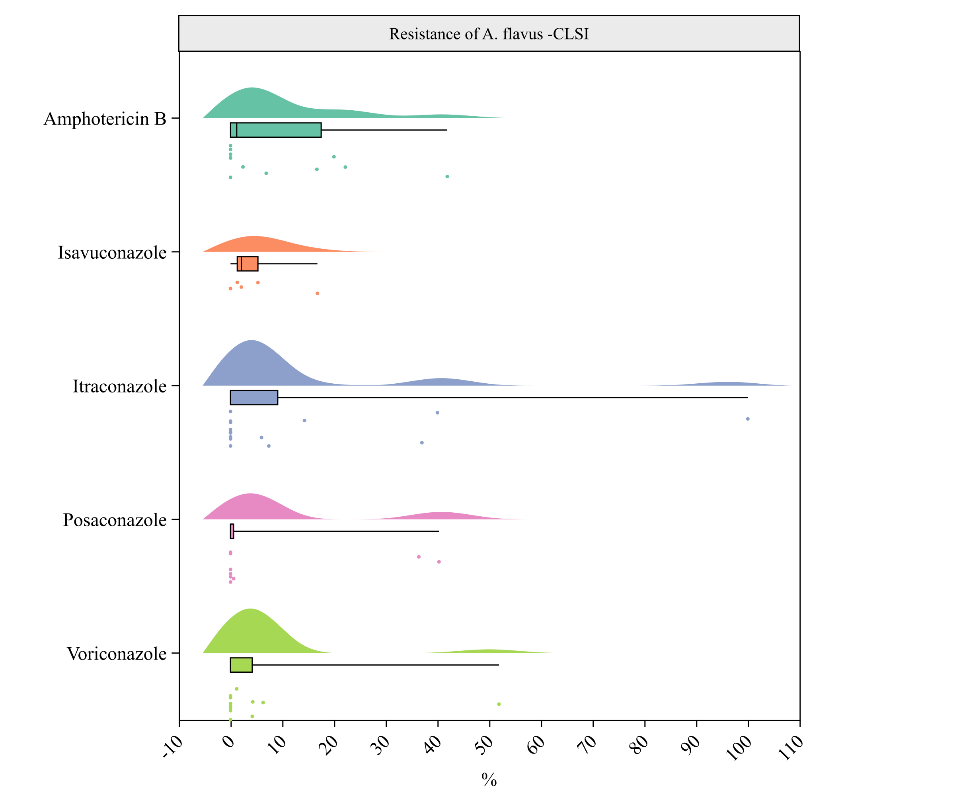 | 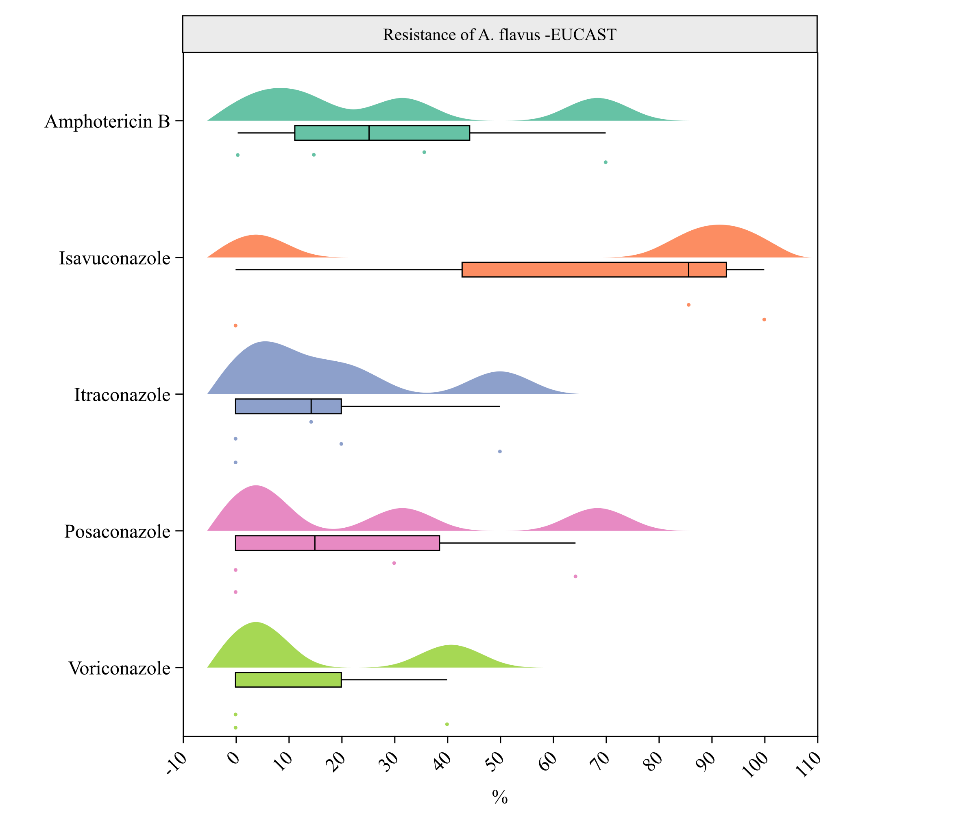 |
| B. *A.* *fumigatus* | 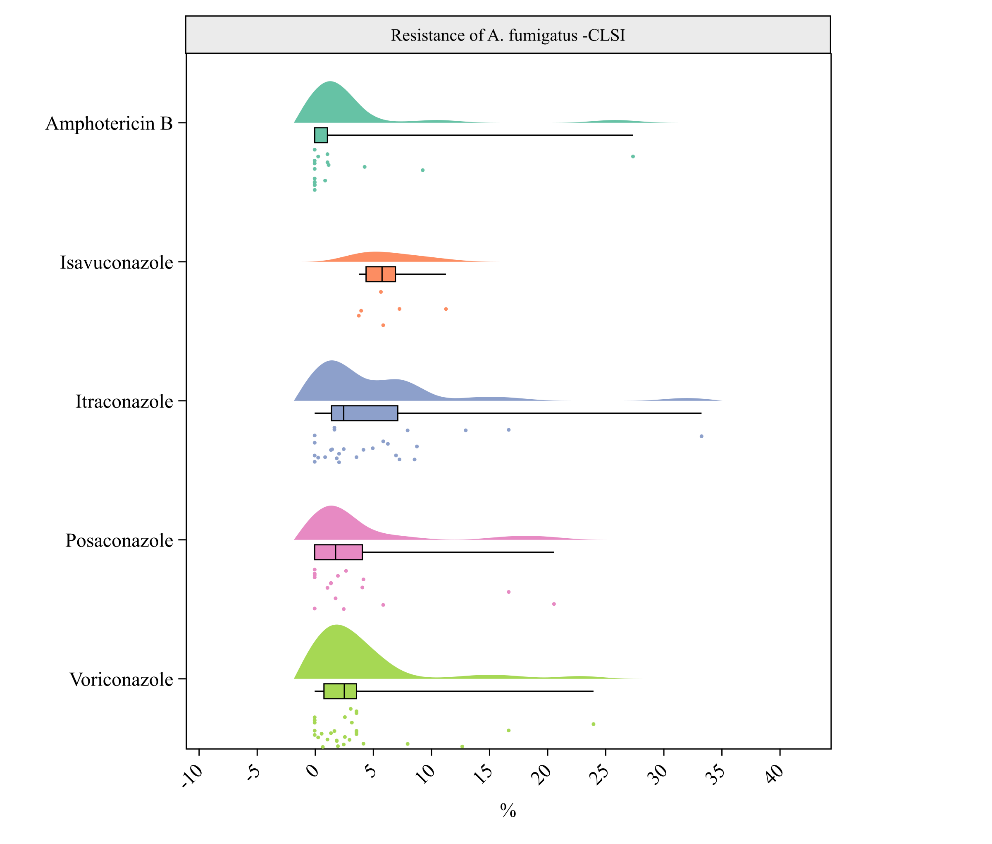 | 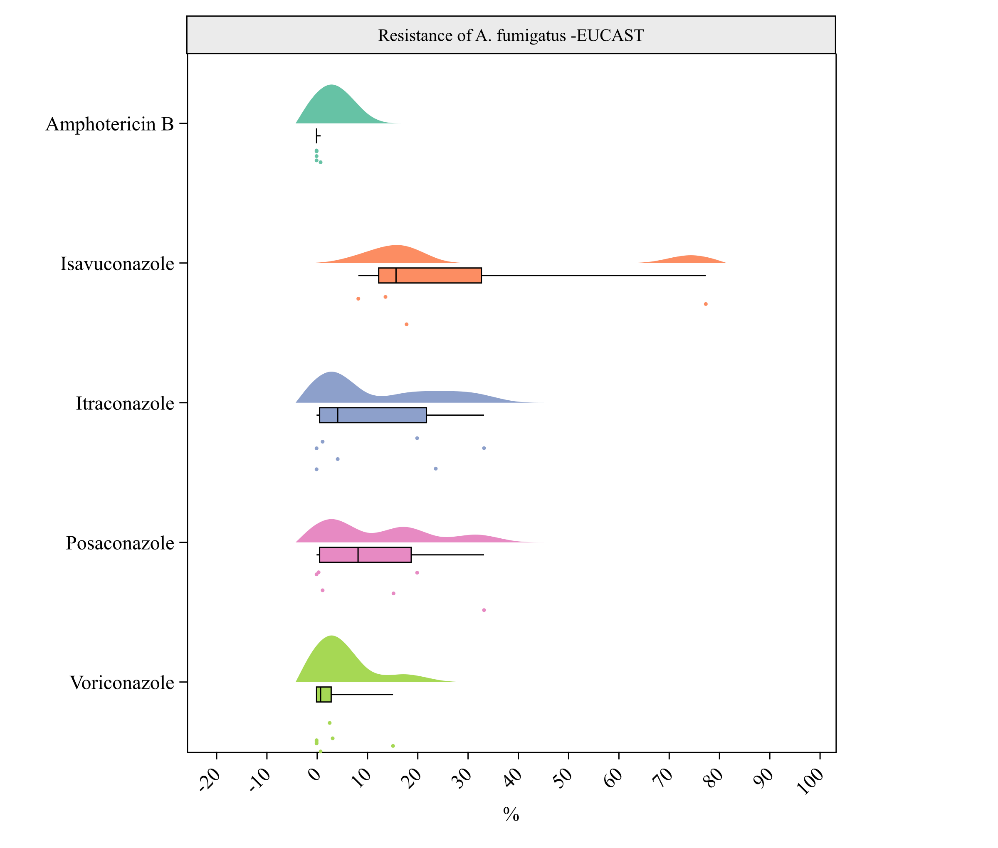 |
| C. *A.* *niger* | 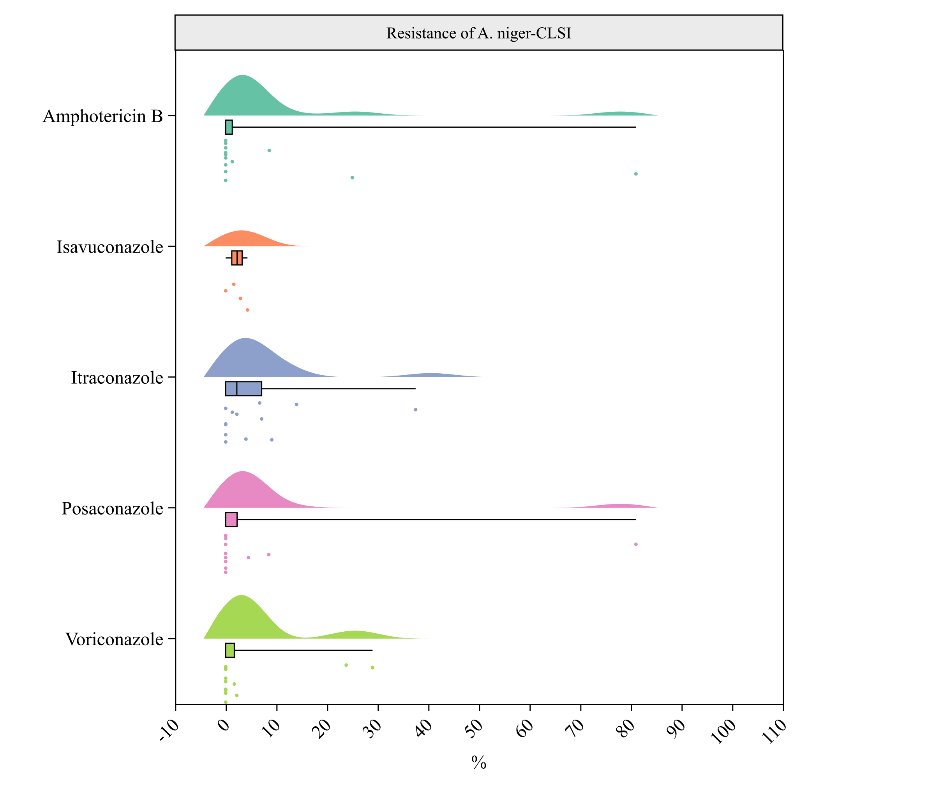 | 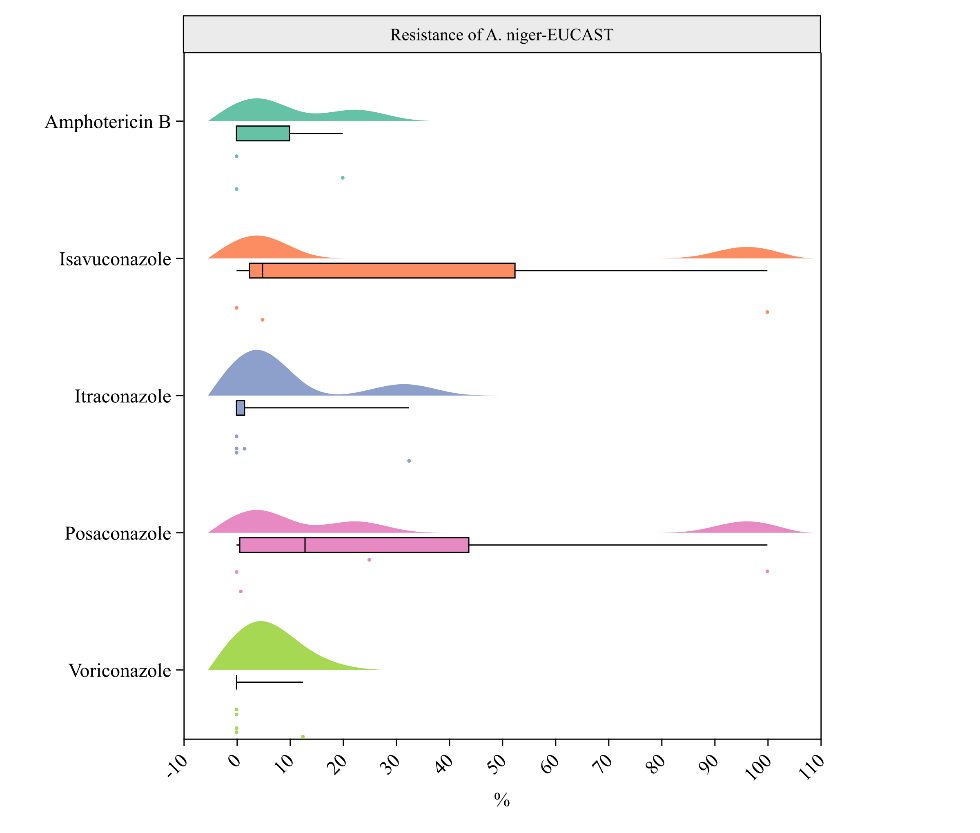 |

## Appendix Figure 6. Resistance of *Aspergillus* isolated from patients to antifungal agents

## Appendix Table 1. MIC50 of antifungal agents for *Aspergillus* isolates

| *Aspergillus* isolates | MIC method | Drug | No. of studies | Median (mg/L) | Range (mg/L) | Notes |
| --- | --- | --- | --- | --- | --- | --- |
| *A.* *flavus* | CLSI | Amphotericin B | 21 | 1 | 0.125–4 | – |
|  |  | Isavuconazole | 9 | 1 | 0.5–2 | – |
|  |  | Itraconazole | 20 | 0.375 | 0.12–16 | – |
|  |  | Posaconazole | 15 | 0.25 | 0.12–0.5 | – |
|  |  | Voriconazole | 21 | 0.5 | 0.125–1 | – |
|  | EUCAST | Amphotericin B | 7 | 4 | 1–32 | – |
|  |  | Isavuconazole | 5 | 1 | 0.5–1 | – |
|  |  | Itraconazole | 7 | 0.25 | 0.12–1 | – |
|  |  | Posaconazole | 7 | 0.125 | 0.12–1 | – |
|  |  | Voriconazole | 9 | 0.5 | 0.5–1 | – |
| *A.* *fumigatus* | CLSI | Amphotericin B | 26 | 1 | 0.0625–4 | – |
|  |  | Isavuconazole | 10 | 0.5 | 0.25–1 | – |
|  |  | Itraconazole | 29 | 0.5 | 0.125–1 | MIC50 range of itraconazole for *A. fumigatus* reported to be 0.031–0.25 mg/L in Lei 2011 (not shown in the figure) this study was not shown in the figure |
|  |  | Posaconazole | 25 | 0.25 | 0.03–1 | MIC50 of posaconazole for *A.* *fumigatus* reported to be ≤0.03 mg/L in Arendrup 2016 (not shown in the figure) |
|  |  | Voriconazole | 32 | 0.5 | 0.125–4 | – |
|  | EUCAST | Amphotericin B | 7 | 0.5 | 0.25–2 | – |
|  |  | Isavuconazole | 3 | 1 | 0.5–1 | – |
|  |  | Itraconazole | 4 | 0.375 | 0.125–0.5 | – |
|  |  | Posaconazole | 5 | 0.0925 | 0.06–0.5 | MIC50 of posaconazole for *A.* *fumigatus* reported to be ≤0.03 mg/L in Arendrup 2016 (not shown in the figure) |
|  |  | Voriconazole | 7 | 0.5 | 0.25–4 | – |
| *A.* *niger* | CLSI | Amphotericin B | 19 | 0.5 | 0.0312–8 | MIC50 range of amphotericin B for *A. niger* reported to be 0.5–1 mg/L in Szigeti 2011 (not shown in the figure) |
|  |  | Isavuconazole | 6 | 2 | 1–2 | – |
|  |  | Itraconazole | 17 | 1 | 0.032–4 | – |
|  |  | Posaconazole | 12 | 0.25 | 0.063–2 | – |
|  |  | Voriconazole | 19 | 1 | 0.125–2 | – |
|  | EUCAST | Amphotericin B | 4 | 0.25 | 0.12–0.5 | – |
|  |  | Isavuconazole | 2 | 1.25 | 1–1.5 | – |
|  |  | Itraconazole | 3 | 0.5 | 0.5–1 | – |
|  |  | Posaconazole | 3 | 0.125 | 0.12–1 | – |
|  |  | Voriconazole | 5 | 1 | 0.5–1 | – |

CLSI: Clinical and Laboratory Standards Institute; EUCAST: European Committee on Antimicrobial Susceptibility Testing

## Appendix Table 2. MIC50 of antifungal agents for Mucorales isolates

| Mucorales isolates | MIC method | Drug | No. of studies | Median (mg/L) | Range (mg/L) | Notes |
| --- | --- | --- | --- | --- | --- | --- |
| *Mucor* spp. | CLSI | Amphotericin B | 4 | 0.375 | 0.125–0.5 | – |
|  |  | Isavuconazole | 5 | 8 | 4–8 | MIC50 of isavuconazole for *Mucor* spp. reported to be >16 mg/L in Borman 2021 and >8 mg/L in Carvalhaes 2023 (not shown in the figure) |
|  |  | Itraconazole | 5 | 4 | 0.25–4 | – |
|  |  | Posaconazole | 6 | 1 | 0.25–2 | – |
|  |  | Voriconazole | 5 | 16.25 | 0.5–32 | MIC50 of voriconazole for *Mucor* spp. reported to be >16 mg/L in Borman 2021 and >8 mg/L in Carvalhaes 2021 and Carvalhaes 2023 (not shown in the figure) |
| *Rhizomucor* spp. | CLSI | Amphotericin B | 3 | 0.375 | 0.125–0.5 | – |
|  |  | Isavuconazole | 4 | 2 | 2 | – |
|  |  | Itraconazole | 2 | 0.5 | 0.5–1 | – |
|  |  | Posaconazole | 4 | 0.5 | 0.25–0.5 | – |
|  |  | Voriconazole | 2 | – | – | MIC50 of voriconazole for *Rhizomucor* spp. reported to be >16 mg/L in Borman 2021 and >8 mg/L in Carvalhaes 2023 (not shown in the figure) |
| *Rhizopus* spp. | CLSI | Amphotericin B | 5 | 0.5 | 0.25–1 | – |
|  |  | Isavuconazole | 6 | 1 | 0.5–2 | – |
|  |  | Itraconazole | 5 | 1 | 0.25–2 | – |
|  |  | Posaconazole | 6 | 0.5 | 0.25–0.5 | – |
|  |  | Voriconazole | 6 | 8 | 0.5–8 | – |

CLSI: Clinical and Laboratory Standards Institute

## Appendix Table 3. Susceptibility of Aspergillus isolates to antifungal agents

| Aspergillus isolate | MIC method | Drug | No. of studies | Median | Range |
| --- | --- | --- | --- | --- | --- |
| A. flavus | CLSI | Amphotericin B | 12 | 96.75% | 6.5–100% |
|  |  | Isavuconazole | 4 | 97.9% | 83.2–100% |
|  |  | Itraconazole | 10 | 100% | 7.1–100% |
|  |  | Posaconazole | 10 | 97.7% | 59.7–100% |
|  |  | Voriconazole | 10 | 100% | 95.8–100% |
|  | EUCAST | Amphotericin B | 3 | 96% | 30–100% |
|  |  | Isavuconazole | 2 | 98.45% | 98–98.9% |
|  |  | Itraconazole | 4 | 98.55% | 80–100% |
|  |  | Posaconazole | 4 | 99.75% | 35–100% |
|  |  | Voriconazole | 3 | 100% | 60–100% |
| A. fumigatus | CLSI | Amphotericin B | 12 | 100% | 95.7–100% |
|  |  | Isavuconazole | 5 | 95.1% | 88.7–96.2% |
|  |  | Itraconazole | 15 | 93.7% | 83.3–100% |
|  |  | Posaconazole | 10 | 98% | 79.4–100% |
|  |  | Voriconazole | 16 | 96.8% | 83.3–100% |
|  | EUCAST | Amphotericin B | 2 | 100% | 100% |
|  |  | Isavuconazole | 1 | 91.7% | 91.7% |
|  |  | Itraconazole | 3 | 66.7% | 52–100% |
|  |  | Posaconazole | 2 | 33.3% | 33.3% |
|  |  | Voriconazole | 2 | 100% | 100% |
| A. niger | CLSI | Amphotericin B | 6 | 100% | 100% |
|  |  | Isavuconazole | 3 | 97.75% | 95.7–100% |
|  |  | Itraconazole | 7 | 96.9% | 86–100% |
|  |  | Posaconazole | 7 | 100% | 11–100% |
|  |  | Voriconazole | 8 | 98.05% | 71–100% |
|  | EUCAST | Amphotericin B | 1 | 80% | 80% |
|  |  | Isavuconazole | – | – | – |
|  |  | Itraconazole | 1 | 67.5% | 67.5% |
|  |  | Posaconazole | 1 | 50% | 50% |
|  |  | Voriconazole | 1 | 87.5% | 87.5% |

CLSI: Clinical and Laboratory Standards Institute; EUCAST: European Committee on Antimicrobial Susceptibility Testing

## Appendix Table 4. Resistance of *Aspergillus* isolates to antifungal agents

| *Aspergillus* isolate | MIC method | Drug | No. of studies | Median | Range |
| --- | --- | --- | --- | --- | --- |
| *A.* *flavus* | CLSI | Amphotericin B | 12 | 0% | 0–41.9% |
|  |  | Isavuconazole | 4 | 1.7% | 0–16.8% |
|  |  | Itraconazole | 15 | 0% | 0–100% |
|  |  | Posaconazole | 12 | 0% | 0–40.3% |
|  |  | Voriconazole | 14 | 0% | 0–51.9% |
|  | EUCAST | Amphotericin B | 7 | 25.7% | 0–70% |
|  |  | Isavuconazole | 4 | 92.85% | 0–100% |
|  |  | Itraconazole | 7 | 20% | 0–100% |
|  |  | Posaconazole | 7 | 30% | 0–100% |
|  |  | Voriconazole | 8 | 0% | 0–40% |
| *A.* *fumigatus* | CLSI | Amphotericin B | 17 | 0% | 0–27.4% |
|  |  | Isavuconazole | 5 | 5.8% | 3.9–11.3% |
|  |  | Itraconazole | 25 | 2.1% | 0–33.3% |
|  |  | Posaconazole | 15 | 1.8% | 0–20.6% |
|  |  | Voriconazole | 25 | 2.6% | 0–16.7% |
|  | EUCAST | Amphotericin B | 6 | 0% | 0–0.8% |
|  |  | Isavuconazole | 4 | 22.6% | 8.3–87.3% |
|  |  | Itraconazole | 8 | 12.1% | 0–54.5% |
|  |  | Posaconazole | 8 | 8.25% | 0–38.2% |
|  |  | Voriconazole | 8 | 0% | 0–15.2% |
| *A.* *niger* | CLSI | Amphotericin B | 17 | 0% | 0–27.4% |
|  |  | Isavuconazole | 5 | 5.8% | 3.9–11.3% |
|  |  | Itraconazole | 25 | 2.1% | 0–33.3% |
|  |  | Posaconazole | 15 | 1.8% | 0–20.6% |
|  |  | Voriconazole | 25 | 2.6% | 0–16.7% |
|  | EUCAST | Amphotericin B | 6 | 0% | 0–0.8% |
|  |  | Isavuconazole | 4 | 22.6% | 8.3–87.3% |
|  |  | Itraconazole | 8 | 12.1% | 0–54.5% |
|  |  | Posaconazole | 8 | 8.25% | 0–38.2% |
|  |  | Voriconazole | 8 | 0% | 0–15.2% |

CLSI: Clinical and Laboratory Standards Institute; EUCAST: European Committee on Antimicrobial Susceptibility Testing

## Appendix Table 5. Resistance of Mucorales isolates to antifungal agents

| Mucorales isolate | MIC method | Study ID | Drug | Resistance |
| --- | --- | --- | --- | --- |
| *Mucor* spp. | CLSI | Borman 2021 | Amphotericin B | 1.0% |
|  |  |  | Isavuconazole | 100.0% |
|  |  |  | Itraconazole | 58.0% |
|  |  |  | Posaconazole | 80.6% |
|  |  |  | Voriconazole | 97.7% |
|  | EUCAST | – | Amphotericin B | – |
|  |  |  | Isavuconazole | – |
|  |  |  | Itraconazole | – |
|  |  |  | Posaconazole | – |
|  |  |  | Voriconazole | – |
| *Rhizomucor* spp. | CLSI | Borman 2021 | Amphotericin B | 0.0% |
|  |  |  | Isavuconazole | 100.0% |
|  |  |  | Itraconazole | 18.8% |
|  |  |  | Posaconazole | 51.4% |
|  |  |  | Voriconazole | 87.5% |
|  | EUCAST | – | Amphotericin B | – |
|  |  |  | Isavuconazole | – |
|  |  |  | Itraconazole | – |
|  |  |  | Posaconazole | – |
|  |  |  | Voriconazole | – |
| *Rhizopus* spp. | CLSI | Borman 2021 | Amphotericin B | 0.8% |
|  |  |  | Isavuconazole | 74.3% |
|  |  |  | Itraconazole | 39.8% |
|  |  |  | Posaconazole | 80.0% |
|  |  |  | Voriconazole | 100.0% |
|  | EUCAST | Drogari-Apiranthitou 2012 | Amphotericin B | 29.4% |
|  |  |  | Posaconazole | 17.6% |

CLSI: Clinical and Laboratory Standards Institute; EUCAST: European Committee on Antimicrobial Susceptibility Testing

## Appendix Table 6. MIC range of antifungal agents for *Aspergillus* isolates

| *Aspergillus* isolate | MIC method | Drug | No. of studies | MIC range (mg/L) |
| --- | --- | --- | --- | --- |
| *A.* *flavus* | CLSI | Amphotericin B | 32 | 0.0125–32 |
|  |  | Isavuconazole | 13 | 0.06–16 |
|  |  | Itraconazole | 31 | 0.03–64 |
|  |  | Posaconazole | 29 | 0.03–4 |
|  |  | Voriconazole | 33 | 0.03–16 |
|  | EUCAST | Amphotericin B | 10 | 0.5–32 |
|  |  | Isavuconazole | 8 | 0.125–16 |
|  |  | Itraconazole | 9 | 0.031–16 |
|  |  | Posaconazole | 9 | 0.03–4 |
|  |  | Voriconazole | 11 | 0.063–16 |
| *A.* *fumigatus* | CLSI | Amphotericin B | 37 | 0.016–8 |
|  |  | Isavuconazole | 12 | 0.06–32 |
|  |  | Itraconazole | 54 | 0.015–128 |
|  |  | Posaconazole | 46 | 0.008–32 |
|  |  | Voriconazole | 62 | 0.03–32 |
|  | EUCAST | Amphotericin B | 8 | 0.015–2 |
|  |  | Isavuconazole | 20 | 0.06–32 |
|  |  | Itraconazole | 54 | 0.015–128 |
|  |  | Posaconazole | 46 | 0.008–32 |
|  |  | Voriconazole | 62 | 0.03–32 |
| *A.* *niger* | CLSI | Amphotericin B | 31 | 0.016–16 |
|  |  | Isavuconazole | 16 | 0.06–16 |
|  |  | Itraconazole | 25 | 0.015–16 |
|  |  | Posaconazole | 18 | 0.002–4 |
|  |  | Voriconazole | 28 | 0.03–16 |
|  | EUCAST | Amphotericin B | 31 | 0.016–16 |
|  |  | Isavuconazole | 16 | 0.06–16 |
|  |  | Itraconazole | 4 | 0.06–16 |
|  |  | Posaconazole | 23 | 0.002–4 |
|  |  | Voriconazole | 6 | 0.25–2 |

CLSI: Clinical and Laboratory Standards Institute; EUCAST: European Committee on Antimicrobial Susceptibility Testing

## Appendix Table 7. MIC range of antifungal agents for Mucorales isolates

| Mucorales isolate | MIC method | Drug | No. of studies | MIC range (mg/L) |
| --- | --- | --- | --- | --- |
| *Mucor* spp. | CLSI | Amphotericin B | 7 | 0.03–8 |
|  |  | Isavuconazole | 6 | 0.5–32 |
|  |  | Itraconazole | 7 | 0.03–32 |
|  |  | Posaconazole | 9 | 0.008–8 |
|  |  | Voriconazole | 9 | 0.031–32 |
|  | EUCAST | Amphotericin B | – | – |
|  |  | Isavuconazole | – | – |
|  |  | Itraconazole | – | – |
|  |  | Posaconazole | – | – |
|  |  | Voriconazole | – | – |
| *Rhizomucor* spp. | CLSI | Amphotericin B | 5 | 0.03–1 |
|  |  | Isavuconazole | 6 | 0.06–8 |
|  |  | Itraconazole | 5 | 0.03–4 |
|  |  | Posaconazole | 7 | 0.03–4 |
|  |  | Voriconazole | 7 | 2–16 |
|  | EUCAST | Amphotericin B | – | – |
|  |  | Isavuconazole | – | – |
|  |  | Itraconazole | – | – |
|  |  | Posaconazole | – | – |
|  |  | Voriconazole | – | – |
| *Rhizopus* spp. | CLSI | Amphotericin B | 9 | 0.032–32 |
|  |  | Isavuconazole | 7 | 0.125–32 |
|  |  | Itraconazole | 7 | 0.031–32 |
|  |  | Posaconazole | 11 | 0.008–32 |
|  |  | Voriconazole | 13 | 0.03–32 |
|  | EUCAST | Amphotericin B | 2 | 0.12–8 |
|  |  | Isavuconazole | – | – |
|  |  | Itraconazole | 2 | 0.25–16 |
|  |  | Posaconazole | – | – |
|  |  | Voriconazole | 2 | 2–16 |

CLSI: Clinical and Laboratory Standards Institute; EUCAST: European Committee on Antimicrobial Susceptibility Testing

## Appendix Table 8. MIC50 of antifungal agents for Aspergillus isolated from patients

| *Aspergillus* isolate | MIC method | Drug | No. of studies | Median (mg/L) | Range (mg/L) | Notes |
| --- | --- | --- | --- | --- | --- | --- |
| *A.* *flavus* in patients | CLSI | Amphotericin B | 19 | 2 | 0.125–4 | – |
|  |  | Isavuconazole | 12 | 1 | 0.5–2 | – |
|  |  | Itraconazole | 18 | 0.5 | 0.125–16 | – |
|  |  | Posaconazole | 19 | 0.25 | 0.125–0.5 | – |
|  |  | Voriconazole | 19 | 0.5 | 0.125–1 | – |
|  | EUCAST | Amphotericin B | 6 | 2.5 | 1–32 | – |
|  |  | Isavuconazole | 2 | 1 | 1–1 | – |
|  |  | Itraconazole | 3 | 0.25 | 0.12–1 | – |
|  |  | Posaconazole | 1 | 0.125 | 0.12–1 | – |
|  |  | Voriconazole | 7 | 0.5 | 0.5–1 | – |
| *A.* *fumigatus* in patients | CLSI | Amphotericin B | 24 | 1 | 0.0625–4 | – |
|  |  | Isavuconazole | 14 | 0.5 | 0.25–1 | – |
|  |  | Itraconazole | 34 | 0.5 | 0.125–1 | MIC50 range of itraconazole for *A.* *fumigatus* reported to be 0.031–0.25 mg/L in Lei 2011 (not shown in the figure) |
|  |  | Posaconazole | 31 | 0.25 | 0.03–1 | – |
|  |  | Voriconazole | 38 | 0.5 | 0.25–4 | – |
|  | EUCAST | Amphotericin B | 6 | 0.5 | 0.25–3.12 | – |
|  |  | Isavuconazole | 3 | 1 | 1–1 | – |
|  |  | Itraconazole | 2 | 0.5 | 0.12–1 | – |
|  |  | Posaconazole | 5 | 0.125 | 0.06–0.5 | – |
|  |  | Voriconazole | 7 | 0.75 | 0.5–4 | – |
| *A.* *niger* in patients | CLSI | Amphotericin B | 18 | 0.5 | 0.0312–8 | MIC50 range of amphotericin B for *A.* *niger* reported to be 0.5–1 mg/L in Szigeti 2011 (not shown in the figure) |
|  |  | Isavuconazole | 6 | 2 | 1–2 | – |
|  |  | Itraconazole | 16 | 1 | 0.032–4 | – |
|  |  | Posaconazole | 11 | 0.5 | 0.063–2 | – |
|  |  | Voriconazole | 18 | 1 | 0.125–2 | – |
|  | EUCAST | Amphotericin B | 4 | 0.25 | 0.12–6.25 | – |
|  |  | Isavuconazole | 2 | 1.25 | 1–1.5 | – |
|  |  | Itraconazole | 2 | 0.5 | 0.5–1 | – |
|  |  | Posaconazole | 3 | 0.125 | 0.12–1 | – |
|  |  | Voriconazole | 5 | 1 | 0.5–1 | – |

CLSI: Clinical and Laboratory Standards Institute; EUCAST: European Committee on Antimicrobial Susceptibility Testing

## Appendix Table 9. MIC50 of antifungal agents for Mucorales isolated from patients

| Mucorales isolate | MIC method | Drug | No. of studies | Median (mg/L) | Range (mg/L) | Notes |
| --- | --- | --- | --- | --- | --- | --- |
| *Mucor* spp. | CLSI | Amphotericin B | 4 | 0.375 | 0.125–0.5 | – |
|  |  | Isavuconazole | 5 | 8 | 4–8 | MIC50 of isavuconazole for *Mucor* spp. reported to be >16 mg/L in Borman 2021 and >8 mg/L in Carvalhaes 2023 (not shown in the figure) |
|  |  | Itraconazole | 5 | 4 | 0.25–4 | – |
|  |  | Posaconazole | 6 | 1 | 0.25–2 | – |
|  |  | Voriconazole | 5 | 16.25 | 0.5–32 | MIC50 of voriconazole for *Mucor* spp. reported to be >16 mg/L in Borman 2021 and >8 mg/L in Carvalhaes 2021 and Carvalhaes 2023 (not shown in the figure) |
| *Rhizomucor* spp. | CLSI | Amphotericin B | 4 | 0.375 | 0.125–0.5 | – |
|  |  | Isavuconazole | 4 | 2 | 2 | – |
|  |  | Itraconazole | 2 | 1 | 0.5–1 | – |
|  |  | Posaconazole | 4 | 0.5 | 0.25–0.5 | – |
|  |  | Voriconazole | 2 | – | – | MIC50 of voriconazole for *Mucor* spp. reported to be >16 mg/L in Borman 2021 and >8 mg/L in Carvalhaes 2023 (not shown in the figure) |
| *Rhizopus* spp. | CLSI | Amphotericin B | 4 | 0.5 | 0.25–1 | – |
|  |  | Isavuconazole | 5 | 1 | 0.5–2 | – |
|  |  | Itraconazole | 5 | 1 | 0.25–2 | – |
|  |  | Posaconazole | 6 | 0.5 | 0.25–0.5 | – |
|  |  | Voriconazole | 6 | 8 | 0.5–8 | – |

CLSI: Clinical and Laboratory Standards Institute

## Appendix Table 10. Susceptibility of Aspergillus isolated from patients to antifungal agents

| *Aspergillus* isolate | MIC method | Drug | No. of studies | Median | Range |
| --- | --- | --- | --- | --- | --- |
| *A.* *flavus* | CLSI | Amphotericin B | 8 | 98.55% | 6.5–100% |
|  |  | Isavuconazole | 2 | 97.9% | 83.2–100% |
|  |  | Itraconazole | 9 | 100% | 7.1–100% |
|  |  | Posaconazole | 9 | 97.8% | 59.7–100% |
|  |  | Voriconazole | 9 | 100% | 95.8–100% |
|  | EUCAST | Amphotericin B | 1 | 30% | 30–100% |
|  |  | Isavuconazole | – | – | – |
|  |  | Itraconazole | 2 | 90% | 80–100% |
|  |  | Posaconazole | 1 | 100% | 100% |
|  |  | Voriconazole | 1 | 60% | 60% |
| *A.* *fumigatus* | CLSI | Amphotericin B | 11 | 99.85% | 95.7–100% |
|  |  | Isavuconazole | 5 | 95.1% | 88.7–96.2% |
|  |  | Itraconazole | 14 | 93.35% | 83.3–100% |
|  |  | Posaconazole | 10 | 98% | 79.4–100% |
|  |  | Voriconazole | 15 | 96.8% | 91.1–100% |
|  | EUCAST | Amphotericin B | 2 | 100% | 100% |
|  |  | Isavuconazole | 1 | 91.7% | 91.7% |
|  |  | Itraconazole | 3 | 66.7% | 52–100% |
|  |  | Posaconazole | 2 | 33.3% | 33.3% |
|  |  | Voriconazole | 2 | 100% | 100% |
| *A.* *niger* | CLSI | Amphotericin B | 6 | 100.0% | 100.0% |
|  |  | Isavuconazole | 3 | 97.75% | 95.7–100.0% |
|  |  | Itraconazole | 7 | 96.9% | 93.3–100.0% |
|  |  | Posaconazole | 7 | 100.0% | 95.5–100.0% |
|  |  | Voriconazole | 8 | 99.15% | 76.2–100.0% |
|  | EUCAST | Amphotericin B | 1 | 80.0% | 80.0% |
|  |  | Isavuconazole | – | – | – |
|  |  | Itraconazole | 1 | 67.5% | 67.5% |
|  |  | Posaconazole | 1 | 50% | 50% |
|  |  | Voriconazole | 1 | 87.5% | 87.5% |

CLSI: Clinical and Laboratory Standards Institute; EUCAST: European Committee on Antimicrobial Susceptibility Testing

## Appendix Table 11. Resistance of *Aspergillus* isolated from patients to antifungal agents

| *Aspergillus* isolate | MIC method | Drug | No. of studies | Median | Range |
| --- | --- | --- | --- | --- | --- |
| *A.* *flavus* | CLSI | Amphotericin B | 11 | 1.2% | 0–41.9% |
|  |  | Isavuconazole | 4 | 2.1% | 0–16.8% |
|  |  | Itraconazole | 14 | 0% | 0–100% |
|  |  | Posaconazole | 8 | 0% | 0–40.3% |
|  |  | Voriconazole | 12 | 0% | 0–51.9% |
|  | EUCAST | Amphotericin B | 4 | 25.25% | 0.4–70% |
|  |  | Isavuconazole | 2 | 85.7% | 0–100% |
|  |  | Itraconazole | 4 | 14.3% | 0–50% |
|  |  | Posaconazole | 4 | 15% | 0–64.3% |
|  |  | Voriconazole | 3 | 0% | 0–40% |
| *A.* *fumigatus* | CLSI | Amphotericin B | 16 | 0% | 0–27.4% |
|  |  | Isavuconazole | 5 | 5.8% | 3.8–11.3% |
|  |  | Itraconazole | 24 | 2.3% | 0–33.3% |
|  |  | Posaconazole | 15 | 1.8% | 0–20.6% |
|  |  | Voriconazole | 24 | 2.25% | 0–3.6% |
|  | EUCAST | Amphotericin B | 5 | 0% | 0–0.8% |
|  |  | Isavuconazole | 4 | 15.8% | 8.3–77.4% |
|  |  | Itraconazole | 6 | 4.2% | 0–33.3% |
|  |  | Posaconazole | 6 | 8.25% | 0–33.3% |
|  |  | Voriconazole | 6 | 0.8% | 0–15.2% |
| *A.* *niger* | CLSI | Amphotericin B | 11 | 0% | 0–81% |
|  |  | Isavuconazole | 3 | 2.25% | 0–4.3% |
|  |  | Itraconazole | 12 | 2.2% | 0–37.5% |
|  |  | Posaconazole | 10 | 0% | 0–81% |
|  |  | Voriconazole | 12 | 0% | 0–29% |
|  | EUCAST | Amphotericin B | 3 | 0% | 0–20% |
|  |  | Isavuconazole | 2 | 4.9% | 0–100% |
|  |  | Itraconazole | 4 | 0% | 0–32.5% |
|  |  | Posaconazole | 4 | 12.9% | 0–100% |
|  |  | Voriconazole | 4 | 0% | 0–12.5% |

CLSI: Clinical and Laboratory Standards Institute; EUCAST: European Committee on Antimicrobial Susceptibility Testing

## Appendix Table 12. Resistance of Mucorales isolated from patients to antifungal agents

| Mucorales isolate | MIC method | Study ID | Drug | Resistance |
| --- | --- | --- | --- | --- |
| *Mucor* spp. | CLSI | Borman 2021 | Amphotericin B | 1.0% |
|  |  |  | Isavuconazole | 100% |
|  |  |  | Itraconazole | 58.0% |
|  |  |  | Posaconazole | 80.6% |
|  |  |  | Voriconazole | 97.7% |
| *Rhizomucor* spp. | CLSI | Borman 2021 | Amphotericin B | 0.0% |
|  |  |  | Isavuconazole | 100% |
|  |  |  | Itraconazole | 18.8% |
|  |  |  | Posaconazole | 51.4% |
|  |  |  | Voriconazole | 87.5% |
| *Rhizopus* spp. | CLSI | Borman 2021 | Amphotericin B | 0.8% |
|  |  |  | Isavuconazole | 74.3% |
|  |  |  | Itraconazole | 39.8% |
|  |  |  | Posaconazole | 80.0% |
|  |  |  | Voriconazole | 100% |
|  | EUCAST | Drogari-Apiranthitou 2012 | Amphotericin B | 29.4% |
|  |  |  | Posaconazole | 17.6% |

CLSI: Clinical and Laboratory Standards Institute; EUCAST: European Committee on Antimicrobial Susceptibility Testing

## Appendix Table 13. MIC50 of antifungal agents for *Aspergillus* isolated from environment

| *Aspergillus* isolate | MIC method | Study ID | Drug | MIC50 (mg/L) |
| --- | --- | --- | --- | --- |
| *A.* *flavus* | CLSI | Khodavaisy 2016 | Amphotericin B | 0.5 |
|  |  |  | Itraconazole | 0.5 |
|  |  |  | Posaconazole | 1 |
|  |  |  | Voriconazole | 0.25 |
| *A.* *niger* | CLSI | Badali 2016 | Amphotericin B | 1 |
|  |  |  | Itraconazole | 0.5 |
|  |  |  | Posaconazole | 0.063 |
|  |  |  | Voriconazole | 1 |
|  |  | Hivary 2019 | Amphotericin B | 8 |
|  |  |  | Posaconazole | 2 |
|  |  |  | Voriconazole | 1 |

CLSI: Clinical and Laboratory Standards Institute

## Appendix Table 14. Susceptibility of *Aspergillus* isolated from environment to antifungal agents

| *Aspergillus* isolate | MIC method | Study ID | Drug | Susceptibility |
| --- | --- | --- | --- | --- |
| *A.* *flavus* | CLSI | Khodavaisy 2016 | Amphotericin B | 92.8% |
|  |  |  | Itraconazole | 96.4% |
|  |  |  | Posaconazole | 96.4% |
|  |  |  | Voriconazole | 96.4% |
| *A.* *fumigatus* | CLSI | Mohammadi 2016 | Itraconazole | 100.0% |
|  |  | Toyotome 2016 | Amphotericin B | 100.0% |
|  |  |  | Itraconazole | 100.0% |
|  |  |  | Voriconazole | 100.0% |
| *A.* *niger* | CLSI | Hivary 2019 | Amphotericin B | 6.7% |
|  |  |  | Posaconazole | 6.7% |
|  |  |  | Voriconazole | 86.7% |

CLSI: Clinical and Laboratory Standards Institute

## Appendix Table 15. Resistance of *Aspergillus* isolated from environment to antifungal agents

| *Aspergillus* isolate | MIC method | Study ID | Drug | Resistance |
| --- | --- | --- | --- | --- |
| *A.* *flavus* | CLSI | Duong 2020 | Amphotericin B | 25.7% |
|  |  |  | Itraconazole | 48.6% |
|  |  |  | Posaconazole | 77.1% |
|  |  |  | Voriconazole | 17.1% |
|  |  | Monteiro 2019 | Amphotericin B | 66.7% |
|  |  |  | Isavuconazole | 100.0% |
|  |  |  | Itraconazole | 33.3% |
|  |  |  | Posaconazole | 100.0% |
|  |  |  | Voriconazole | 0.0% |
| *A.* *fumigatus* | CLSI | Mohammadi 2016 | Itraconazole | 0.0% |
|  |  |  | Posaconazole | 0.0% |
|  |  |  | Voriconazole | 0.0% |
|  |  | Monteiro 2019 | Amphotericin B | 0.0% |
|  |  |  | Isavuconazole | 87.3% |
|  |  |  | Itraconazole | 21.8% |
|  |  |  | Posaconazole | 38.2% |
|  |  |  | Voriconazole | 0.0% |
| *A.* *niger* | CLSI | Hivary 2019 | Amphotericin B | 93.3% |
|  |  |  | Posaconazole | 93.3% |
|  |  |  | Voriconazole | 13.3% |
|  |  | Monteiro 2019 | Amphotericin B | 0.0% |
|  |  |  | Isavuconazole | 100.0% |
|  |  |  | Itraconazole | 50.0% |
|  |  |  | Posaconazole | 100.0% |
|  |  |  | Voriconazole | 0.0% |

CLSI: Clinical and Laboratory Standards Institute

## Appendix Table 16. Antifungal susceptibility and resistance data (MIC50, MIC range, susceptibility, and resistance) of different strains to antifungal agents in different populations

| Disease | MIC method | Study ID | Strain | Drug | MIC50 (mg/L) | MIC range (mg/L) | Susceptibility | Resistance |
| --- | --- | --- | --- | --- | --- | --- | --- | --- |
| Hematological diseases | CLSI | Colozza 2012 | *A.* *fumigatus* complex | Amphotericin B | 0.25 | 0.25–0.5 | 100.00% | 0.00% |
|  |  |  | *A.* *fumigatus* | Amphotericin B | 0.25 | 0.25–0.5 | 100.00% | 0.00% |
|  |  |  | *A.* *flavus* complex | Amphotericin B | 1 | 0.5–2 | 83.30% | 16.70% |
|  |  |  | *A.* *flavus* | Amphotericin B | 1 | 0.5–2 | 80.00% | 20.00% |
|  |  |  | *A.* *niger* complex | Amphotericin B | — | 0.12–1 | 100.00% | 0.00% |
|  |  |  | *A.* *niger* | Amphotericin B | 0.5 | 0.12–0.5 | 100.00% | 0.00% |
| Invasive fungal infections | CLSI | Carvalhaes 2021 | *A.* *fumigatus* | Isavuconazole | 0.5 | NR | 95.90% | —— |
|  |  |  |  | Itraconazole | 1 | NR | 84.50% | — |
|  |  |  |  | Posaconazole | 0.25 | NR | 97.30% | — |
|  |  |  |  | Voriconazole | 0.5 | NR | 96.80% | — |
|  |  |  | *Rhizopus* spp. | Isavuconazole | 1 | 0.5–2 | — | — |
|  |  |  |  | Itraconazole | 1 | 1–2 | — | — |
|  |  |  |  | Posaconazole | 0.5 | 0.25–1 | — | — |
|  |  |  |  | Voriconazole | 1 | 4–8 | — | — |
|  |  |  | *Mucor* spp. | Isavuconazole | 4 | 2–8 | — | — |
|  |  |  |  | Itraconazole | 4 | 2–8 | — | — |
|  |  |  |  | Posaconazole | 1 | 1–8 | — | — |
|  |  |  |  | Voriconazole | ＞8 | ＞8 | — | — |
|  |  |  | *Rhizomucor* spp. | Isavuconazole | — | 1 | — | — |
|  |  |  |  | Itraconazole | — | 1 | — | — |
|  |  |  |  | Posaconazole | — | 0.5 | — | — |
|  |  |  |  | Voriconazole | — | 8 | — | — |
|  |  | Pfaller 2015 | *A.* *fumigatus* | Amphotericin B | 1 | 1 | — | — |
|  |  |  |  | Itraconazole | 1 | 0.5–1 | 100.00% | 0.00% |
|  |  |  |  | Posaconazole | 0.5 | 0.25–0.5 | 100.00% | 0.00% |
|  |  |  |  | Voriconazole | 0.25 | 0.25–0.5 | 100.00% | 0.00% |
|  |  |  | *A.* *flavus* | Amphotericin B | 2 | 1–2 | — | — |
|  |  |  |  | Itraconazole | 0.5 | 0.5–1 | 83.30% | 16.70% |
|  |  |  |  | Posaconazole | 0.5 | 0.25–0.5 | 83.30% | 16.70% |
|  |  |  |  | Voriconazole | 0.5 | 0.25–1 | 83.30% | 16.70% |
|  |  | Pfaller 2011 | *A.* *fumigatus* | Itraconazole | 0.5 | 0.5–1 | — | 0.00% |
|  |  |  |  | Posaconazole | 0.25 | 0.25–0.5 | — | 0.00% |
|  |  |  |  | Voriconazole | 0.25 | 0.25–0.5 | — | 0.00% |
|  |  | Sarigüzel 2023 | *A.* *fumigatus* | Amphotericin B | 0.125 | 0.016–1.5 | 95.70% | 4.30% |
|  |  |  |  | Itraconazole | 1 | 0.125–1 | 100.00% | — |
|  |  |  |  | Posaconazole | 0.032 | 0.002–0.25 | 100.00% | — |
|  |  |  |  | Voriconazole | 0.125 | 0.064–0.25 | 100.00% | — |
|  |  |  | *A.* *niger* | Amphotericin B | 0.125 | 0.125–0.5 | 100.00% | — |
|  |  |  |  | Itraconazole | 1 | 0.125–1 | 100.00% | — |
|  |  |  |  | Posaconazole | 0.064 | 0.002–0.25 | 100.00% | — |
|  |  |  |  | Voriconazole | 0.125 | 0.036–0.25 | 100.00% | — |
|  |  |  | *A.* *flavus* | Amphotericin B | 0.5 | 0.25–0.5 | 100.00% | — |
|  |  |  |  | Itraconazole | 0.25 | 0.25–1 | 100.00% | — |
|  |  |  |  | Posaconazole | 0.125 | 0.032–0.25 | 100.00% | — |
|  |  |  |  | Voriconazole | 0.125 | 0.064–0.5 | 100.00% | — |
| Aspergillosis | CLSI | Erami 2023 | *A.* *fumigatus* | Amphotericin B | — | 0.125–1 | 100.00% | — |
|  |  |  |  | Itraconazole | — | 0.03–16 | 66.70% | 33.30% |
|  |  |  |  | Voriconazole | — | 0.03–1 | 100.00% | — |
|  |  |  | *A.* *flavus* | Amphotericin B | — | 0.25–1 | 100.00% | — |
|  |  |  |  | Itraconazole | — | 0.25–16 | 60.00% | 40.00% |
|  |  |  |  | Voriconazole | — | 0.125–1 | 100.00% | — |
|  |  | Escribano 2013 | *A.* *fumigatus* | Itraconazole | — | 0.25–4 | — | 0.30% |
|  |  |  |  | Posaconazole | — | 0.06–1 | — | 1.80% |
|  |  |  |  | Voriconazole | — | 0.25–16 | — | 0.30% |
|  |  |  | *A.* *fumigatus* complex | Itraconazole | — | 0.25–16 | — | 2.50% |
|  |  |  |  | Posaconazole | — | 0.06–1 | — | 4.20% |
|  |  |  |  | Voriconazole | — | 0.25–16 | — | 3.10% |
| Aspergillosis | CLSI | Erami 2023 | *A.* *fumigatus* | Amphotericin B | — | 0.125–1 | 100.00% | — |
|  |  |  |  | Itraconazole | — | 0.03–16 | 66.70% | 33.30% |
|  |  |  |  | Voriconazole | — | 0.03–1 | 100.00% | — |
|  |  |  | *A.* *flavus* | Amphotericin B | — | 0.25–1 | 100.00% | — |
|  |  |  |  | Itraconazole | — | 0.25–16 | 60.00% | 40.00% |
|  |  |  |  | Voriconazole | — | 0.125–1 | 100.00% | — |
|  |  | Escribano 2013 | *A.* *fumigatus* | Itraconazole | — | 0.25–4 | — | 0.30% |
|  |  |  |  | Posaconazole | — | 0.06–1 | — | 1.80% |
|  |  |  |  | Voriconazole | — | 0.25–16 | — | 0.30% |
|  |  |  | *A.* *fumigatus* complex | Itraconazole | — | 0.25–16 | — | 2.50% |
|  |  |  |  | Posaconazole | — | 0.06–1 | — | 4.20% |
|  |  |  |  | Voriconazole | — | 0.25–16 | — | 3.10% |
